# Supplementary figures and images for: The myocardium utilizes a platelet-derived growth factor receptor alpha (Pdgfra)–phosphoinositide 3-kinase (PI3K) signaling cascade to steer toward the midline during zebrafish heart tube formation
Source: eLife. 2023 Nov 3;12:e85930. doi: 10.7554/eLife.85930 (PMC10651176; doi:10.7554/eLife.85930)

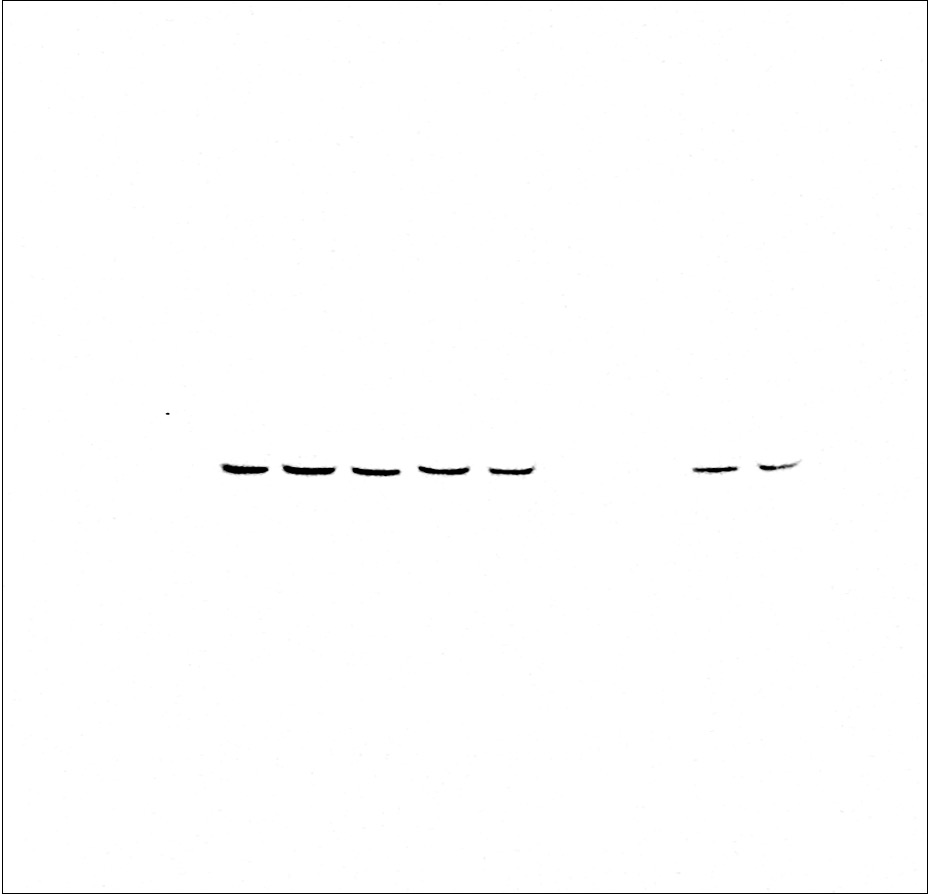

Supplement: Figure 1—source data 2. [file elife-85930-fig1-data2.zip › Figure 1 - source data 2 /Figure 1H representative blots - raw/Akt.jpg]

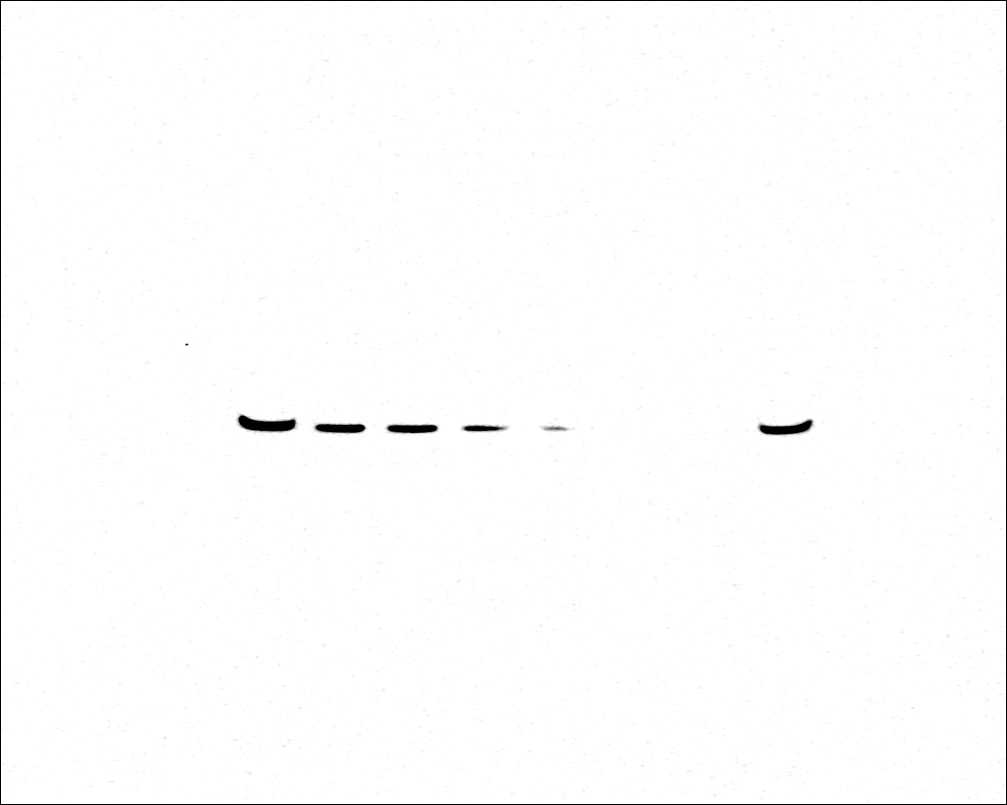

Supplement: Figure 1—source data 2. [file elife-85930-fig1-data2.zip › Figure 1 - source data 2 /Figure 1H representative blots - raw/pAkt.jpg]

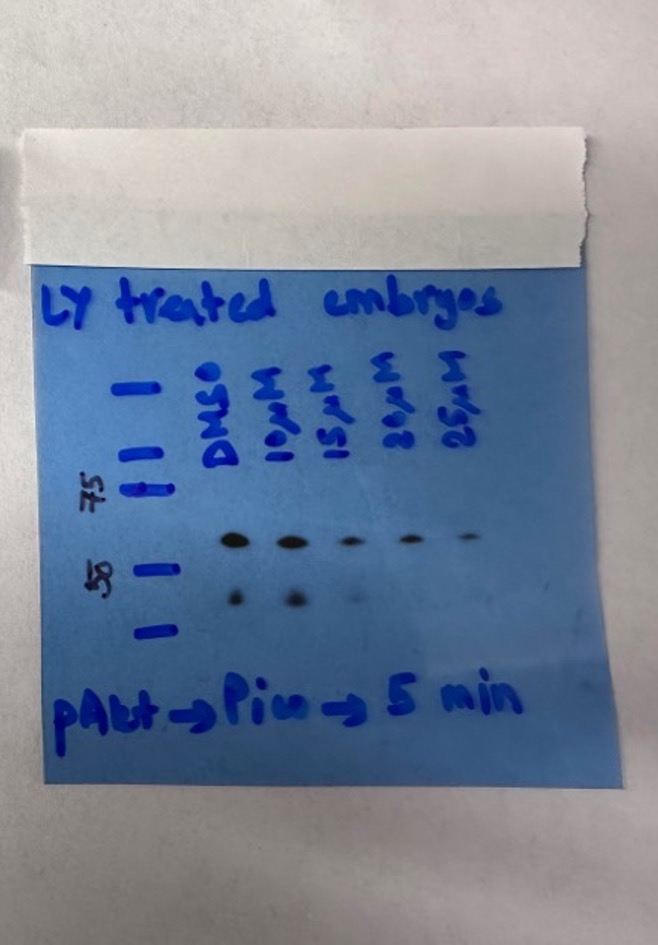

Supplement: Figure 1—source data 2. [file elife-85930-fig1-data2.zip › Figure 1 - source data 2 /Blots used for Figure 1H graph- raw/pAkt_2nd replicate.jpg]

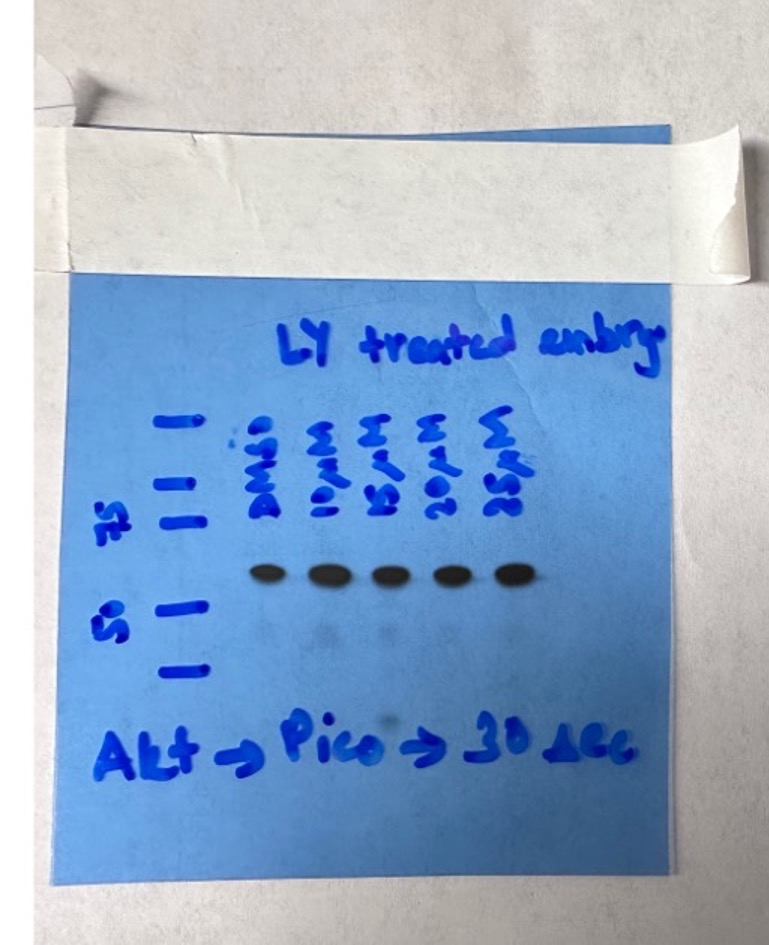

Supplement: Figure 1—source data 2. [file elife-85930-fig1-data2.zip › Figure 1 - source data 2 /Blots used for Figure 1H graph- raw/Akt_2nd replicate.jpg]

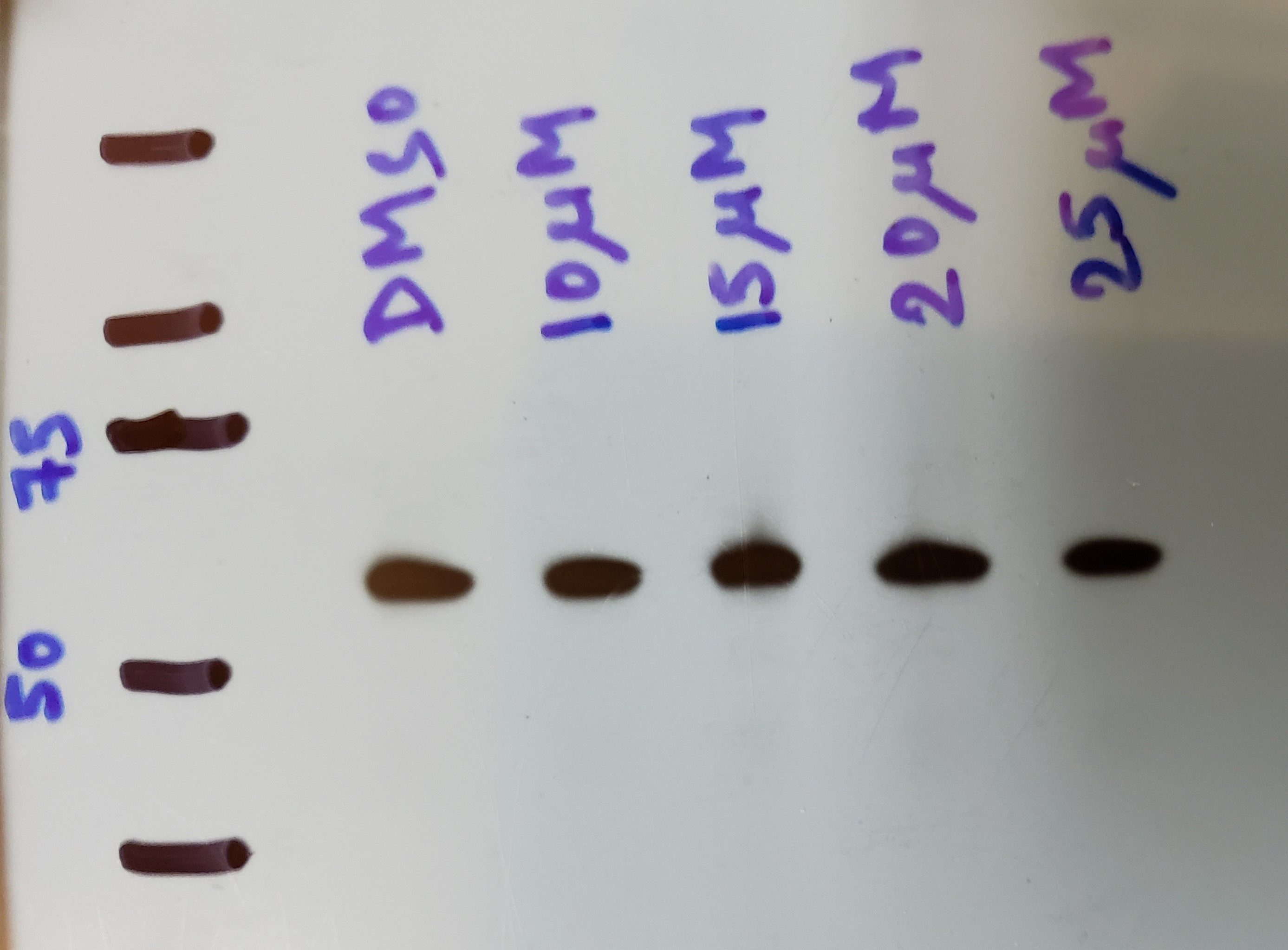

Supplement: Figure 1—source data 2. [file elife-85930-fig1-data2.zip › Figure 1 - source data 2 /Blots used for Figure 1H graph- raw/Akt_1st replicate.jpg]

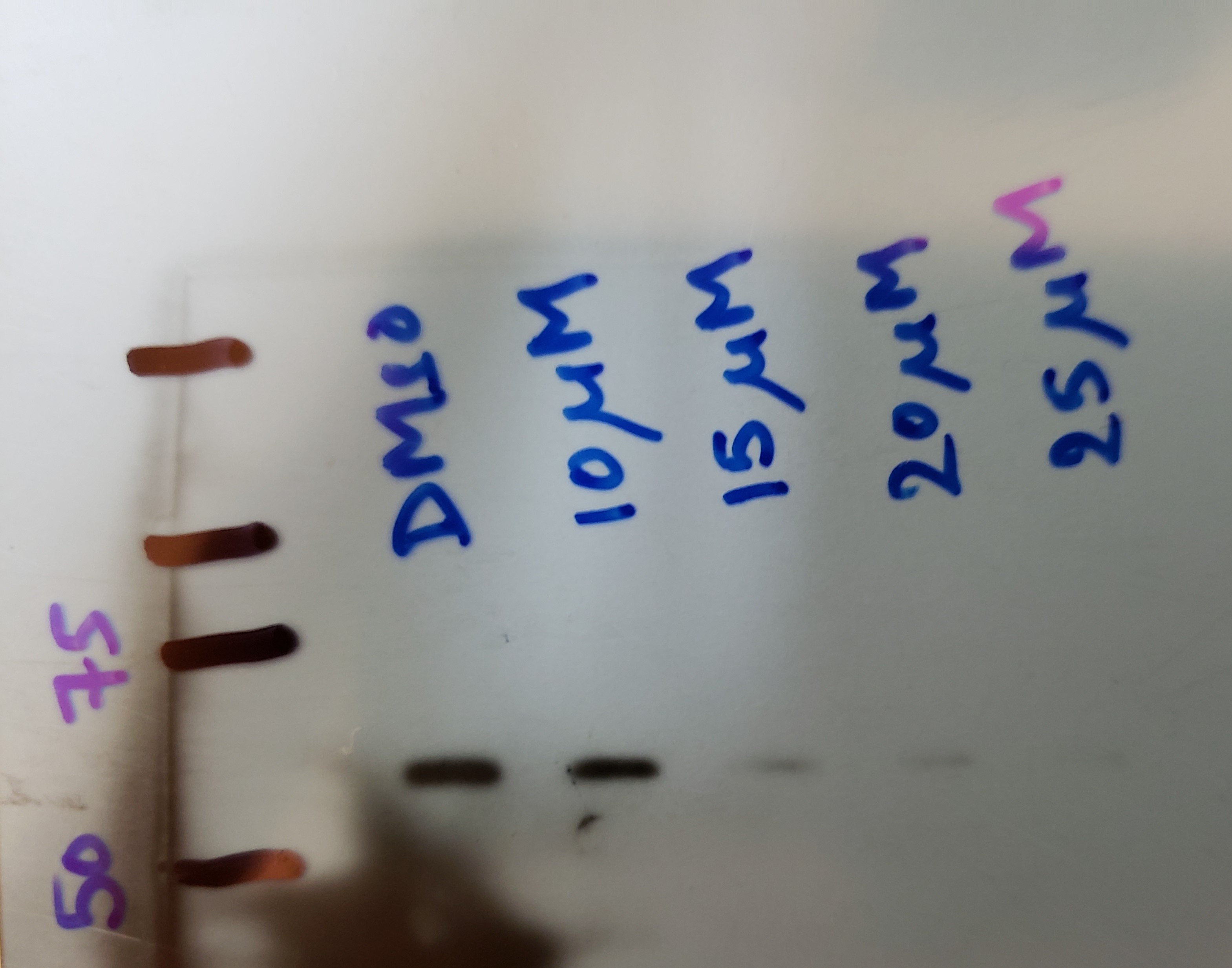

Supplement: Figure 1—source data 2. [file elife-85930-fig1-data2.zip › Figure 1 - source data 2 /Blots used for Figure 1H graph- raw/pAkt_1st replicate.jpg]

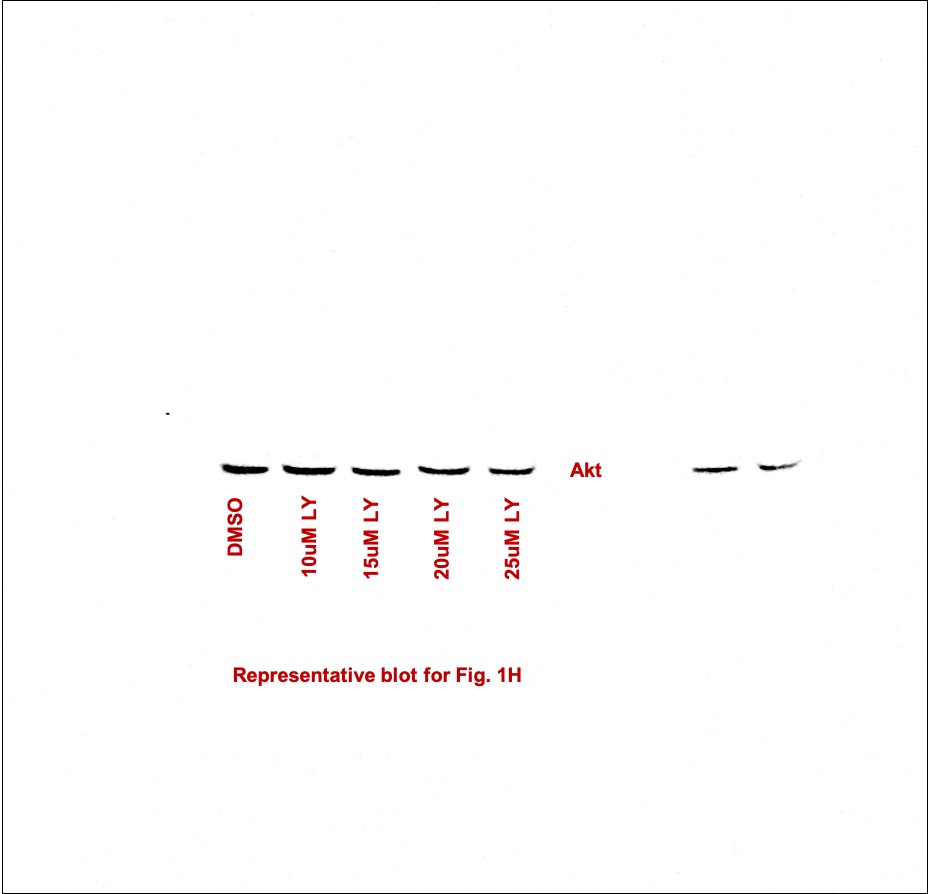

Supplement: Figure 1—source data 2. [file elife-85930-fig1-data2.zip › Figure 1 - source data 2 /Figure 1H representative blots- labelled/Akt.jpg]

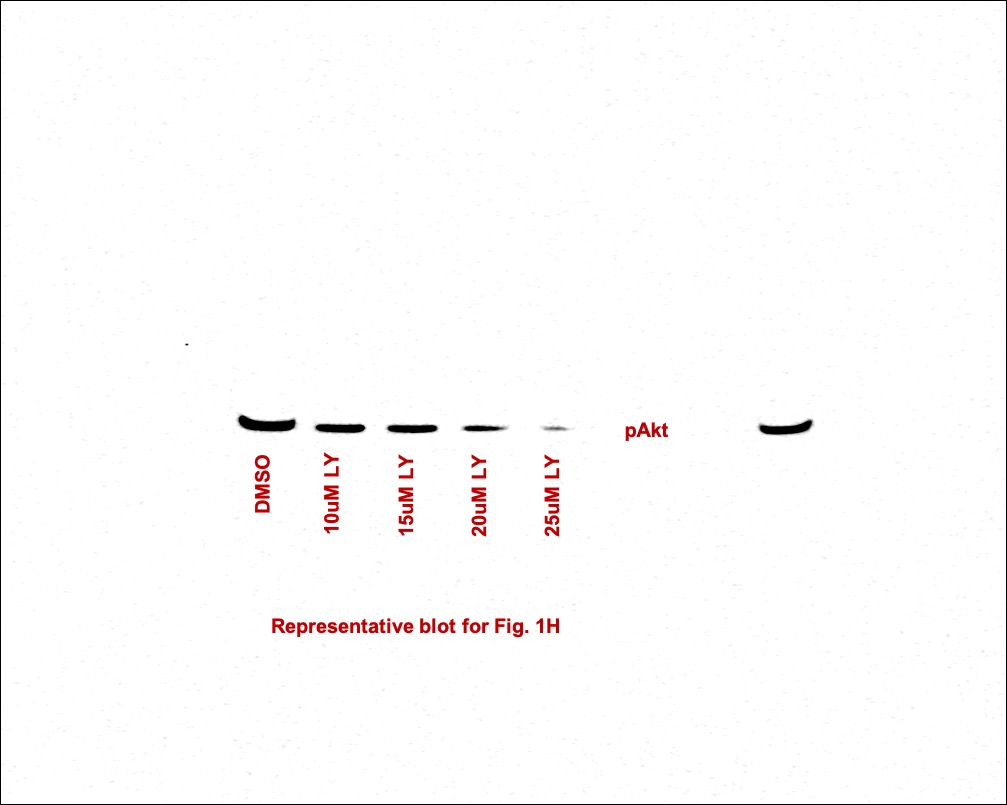

Supplement: Figure 1—source data 2. [file elife-85930-fig1-data2.zip › Figure 1 - source data 2 /Figure 1H representative blots- labelled/pAkt.jpg]

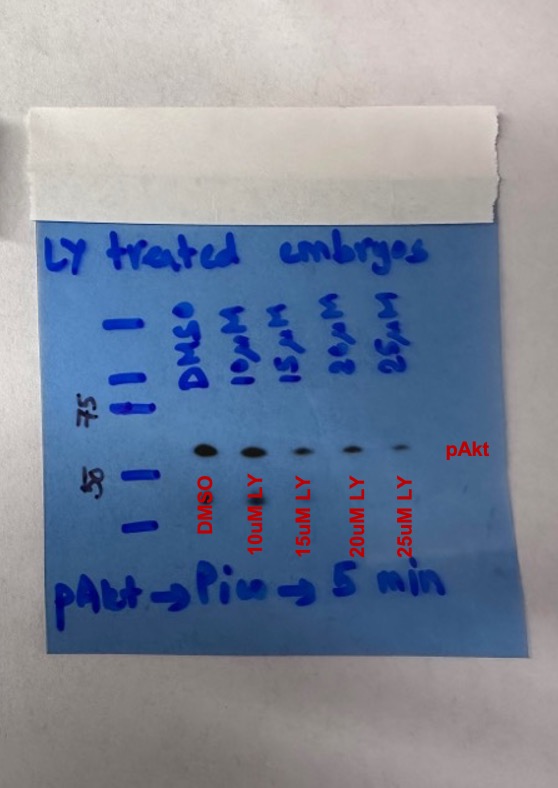

Supplement: Figure 1—source data 2. [file elife-85930-fig1-data2.zip › Figure 1 - source data 2 /Blots used for Figure 1H graph- labelled/pAkt_2nd replicate.jpg]

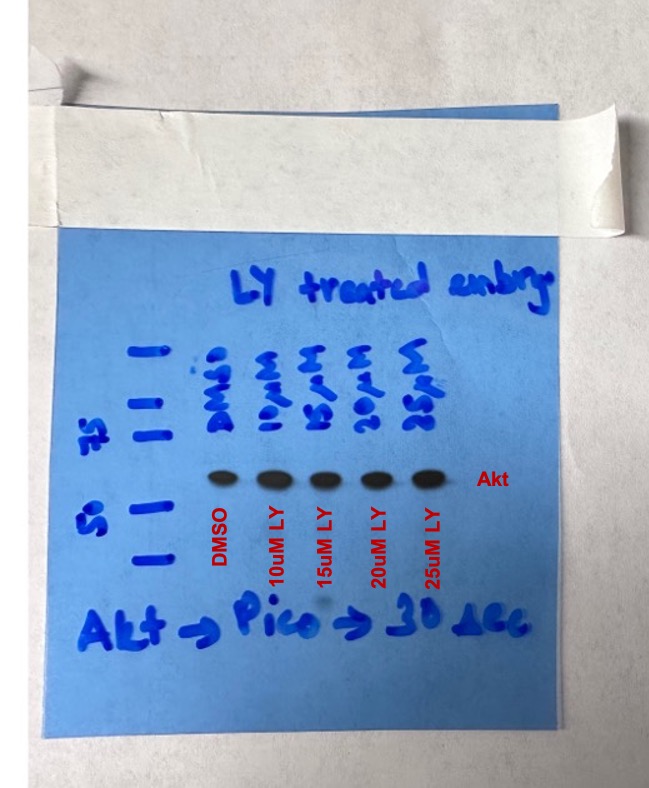

Supplement: Figure 1—source data 2. [file elife-85930-fig1-data2.zip › Figure 1 - source data 2 /Blots used for Figure 1H graph- labelled/Akt_2nd replicate.jpg]

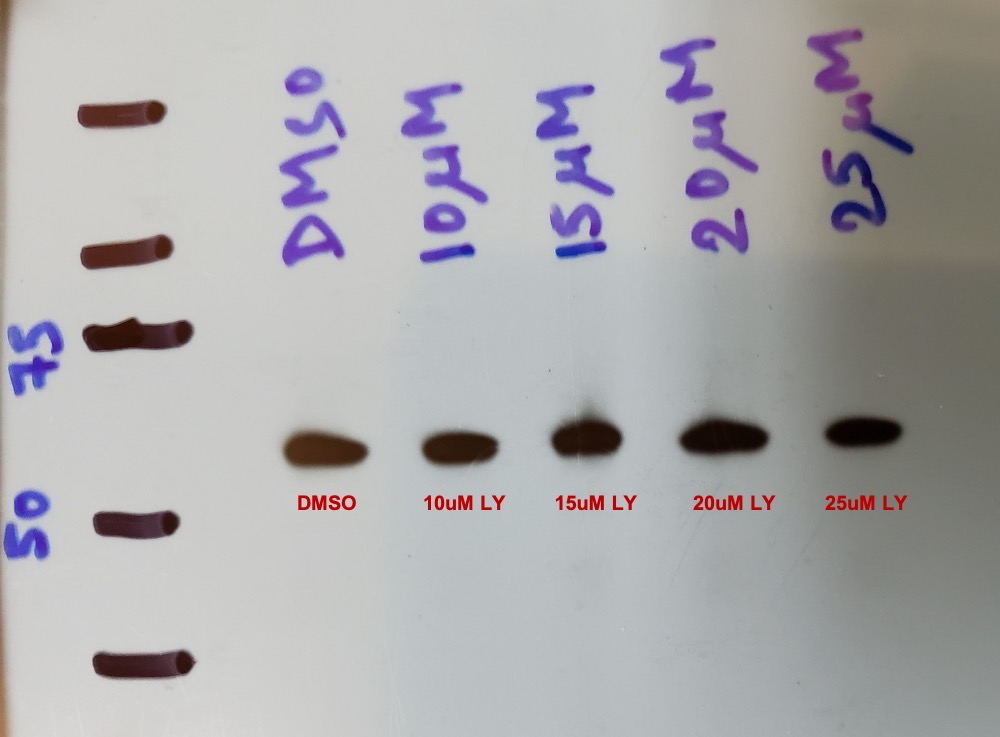

Supplement: Figure 1—source data 2. [file elife-85930-fig1-data2.zip › Figure 1 - source data 2 /Blots used for Figure 1H graph- labelled/Akt_1st replicate.jpg]

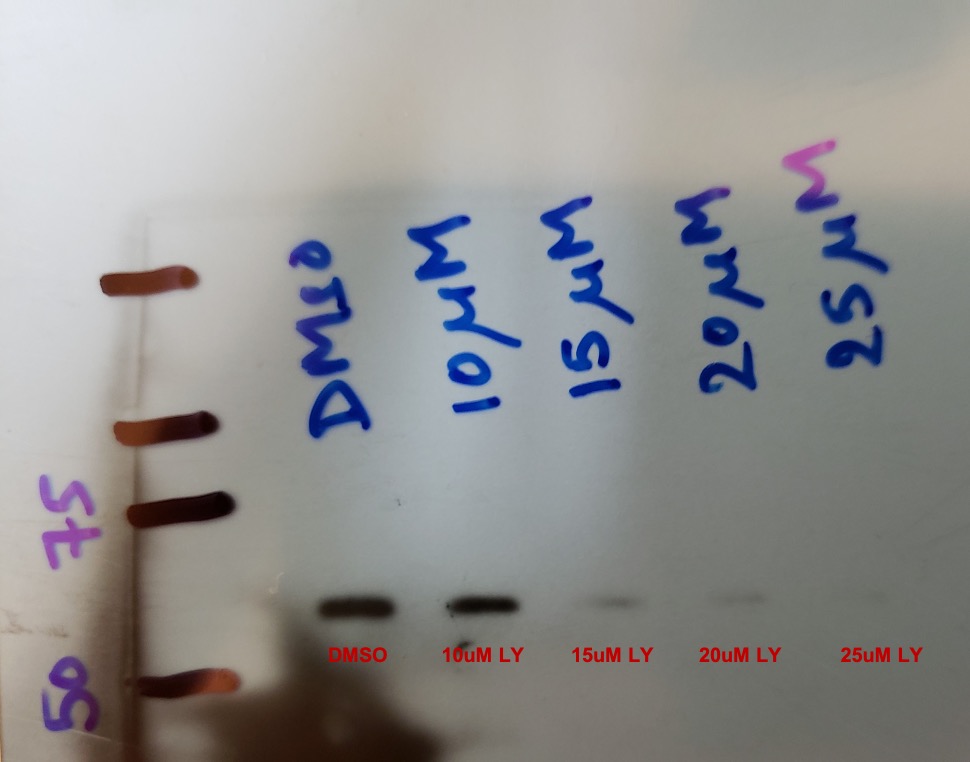

Supplement: Figure 1—source data 2. [file elife-85930-fig1-data2.zip › Figure 1 - source data 2 /Blots used for Figure 1H graph- labelled/pAkt_1st replicate.jpg]

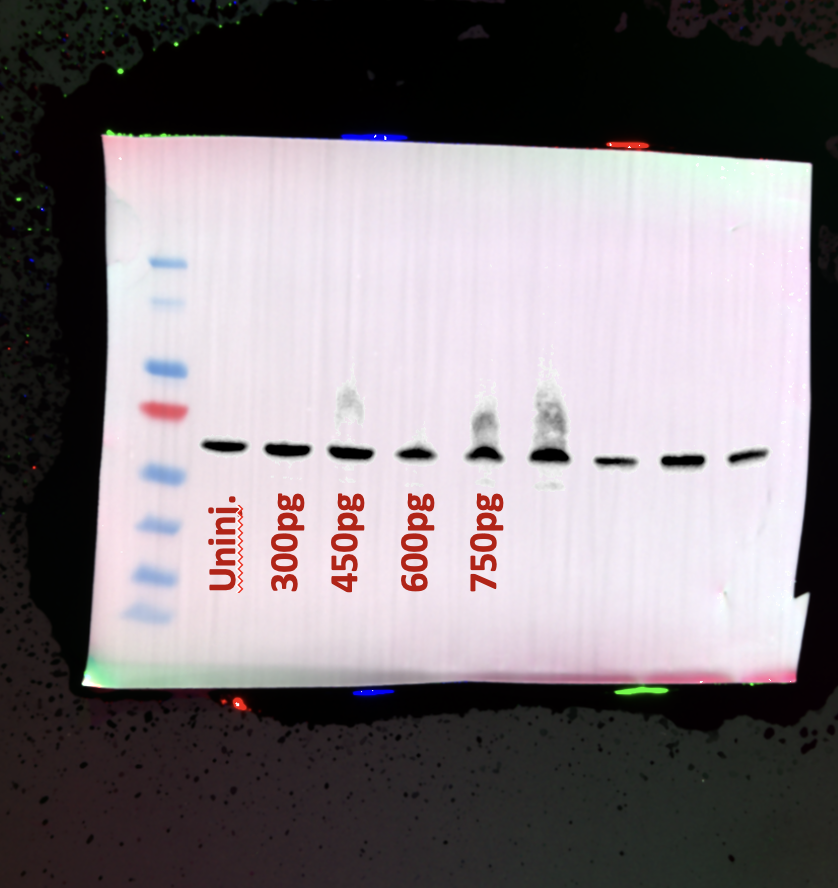

Supplement: Figure 1—figure supplement 1—source data 2. [file elife-85930-fig1-figsupp1-data2.zip › Figure 1 - Figure supplement 1- source data 2/Figure 1 - Figure Supplement 1S/Figure 1 - Figure Supplement 1S representative blots- labelled/dnPI3K_Akt_2nd replicate.tiff]

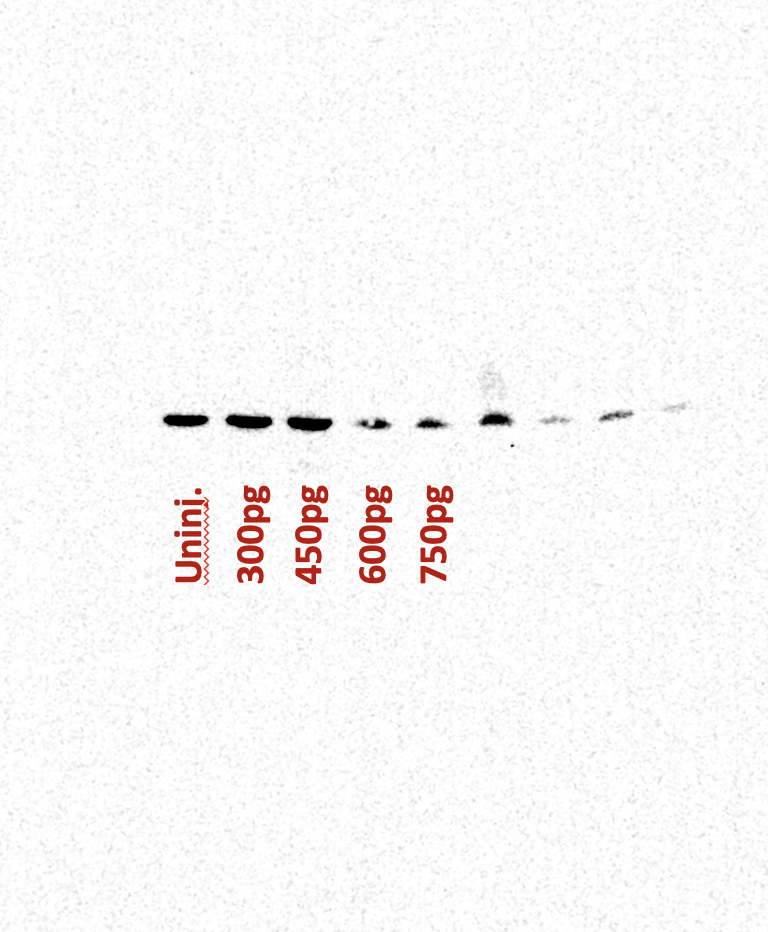

Supplement: Figure 1—figure supplement 1—source data 2. [file elife-85930-fig1-figsupp1-data2.zip › Figure 1 - Figure supplement 1- source data 2/Figure 1 - Figure Supplement 1S/Figure 1 - Figure Supplement 1S representative blots- labelled/dnPI3K_pAkt_2nd replicate_labelled.tiff]

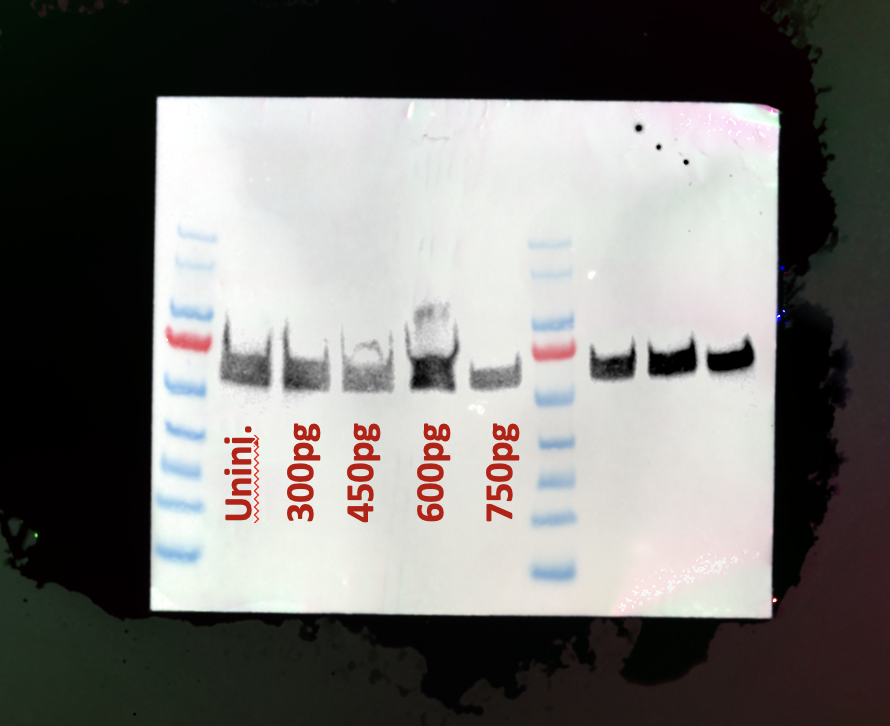

Supplement: Figure 1—figure supplement 1—source data 2. [file elife-85930-fig1-figsupp1-data2.zip › Figure 1 - Figure supplement 1- source data 2/Figure 1 - Figure Supplement 1S/Blots used for Figure 1 - Figure Supplement 1S graph- labelled/dnPI3K_Akt_3rd replicate_labelled.tiff]

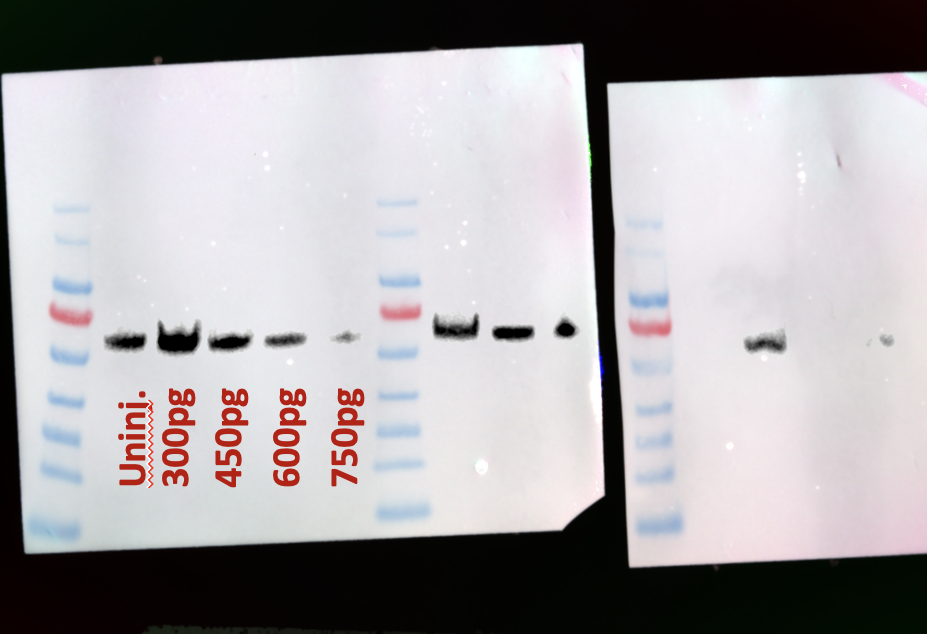

Supplement: Figure 1—figure supplement 1—source data 2. [file elife-85930-fig1-figsupp1-data2.zip › Figure 1 - Figure supplement 1- source data 2/Figure 1 - Figure Supplement 1S/Blots used for Figure 1 - Figure Supplement 1S graph- labelled/dnPI3K_pAkt_1st replicate_labelled.tiff]

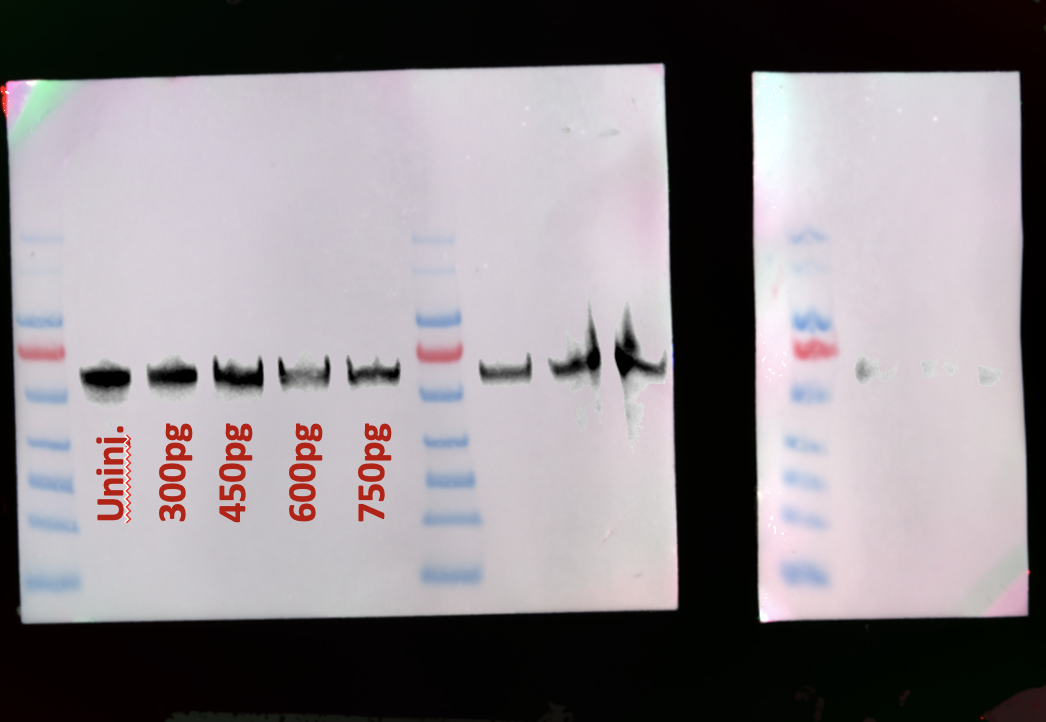

Supplement: Figure 1—figure supplement 1—source data 2. [file elife-85930-fig1-figsupp1-data2.zip › Figure 1 - Figure supplement 1- source data 2/Figure 1 - Figure Supplement 1S/Blots used for Figure 1 - Figure Supplement 1S graph- labelled/dnPI3K_Akt_1st replicate_labelled.tiff]

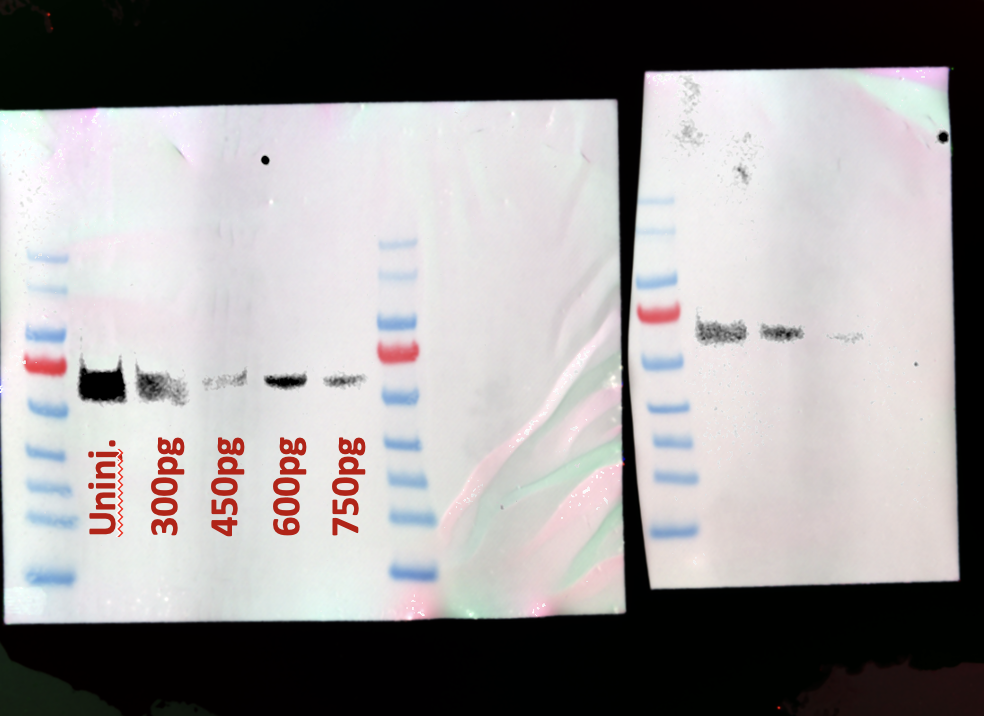

Supplement: Figure 1—figure supplement 1—source data 2. [file elife-85930-fig1-figsupp1-data2.zip › Figure 1 - Figure supplement 1- source data 2/Figure 1 - Figure Supplement 1S/Blots used for Figure 1 - Figure Supplement 1S graph- labelled/dnPI3K_pAkt_3rd replicate_labelled.tiff]

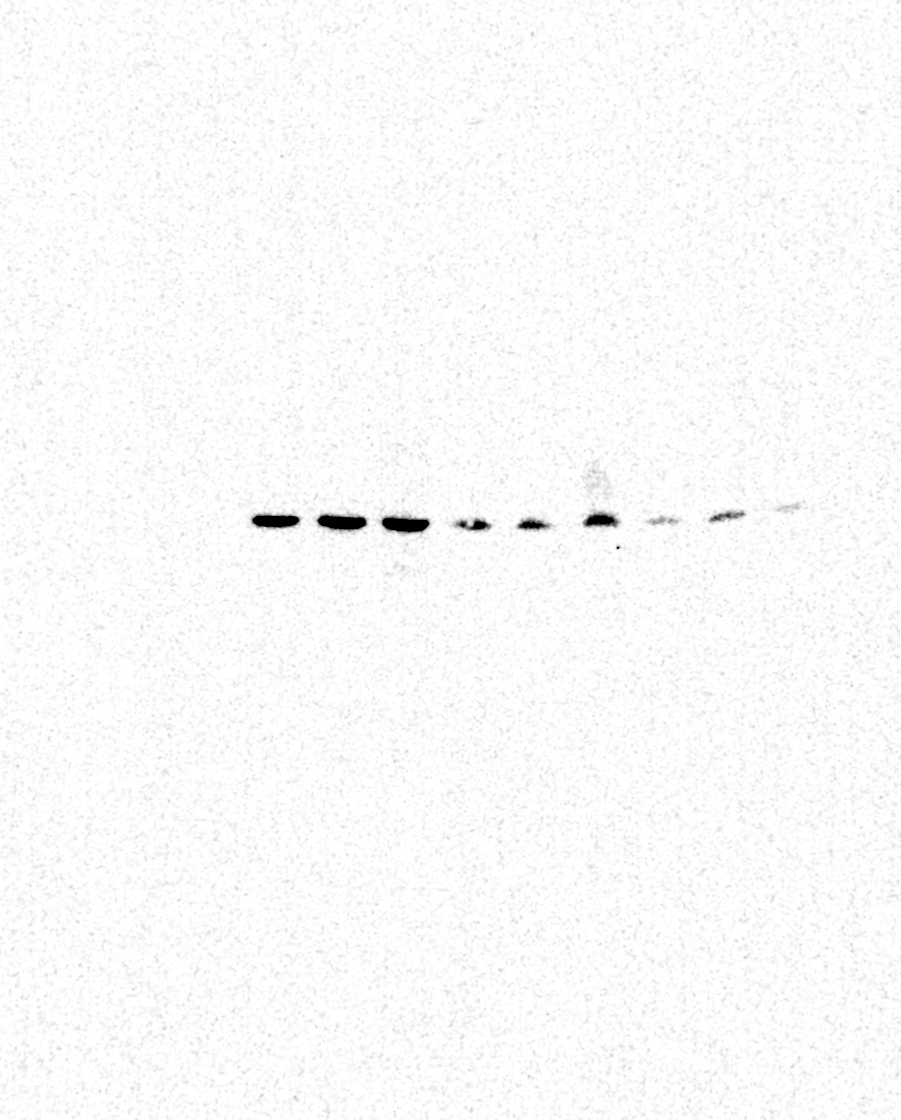

Supplement: Figure 1—figure supplement 1—source data 2. [file elife-85930-fig1-figsupp1-data2.zip › Figure 1 - Figure supplement 1- source data 2/Figure 1 - Figure Supplement 1S/Figure 1 - Figure Supplement 1S representative blots - raw/dnPI3K_pAkt_2nd replicate.tif]

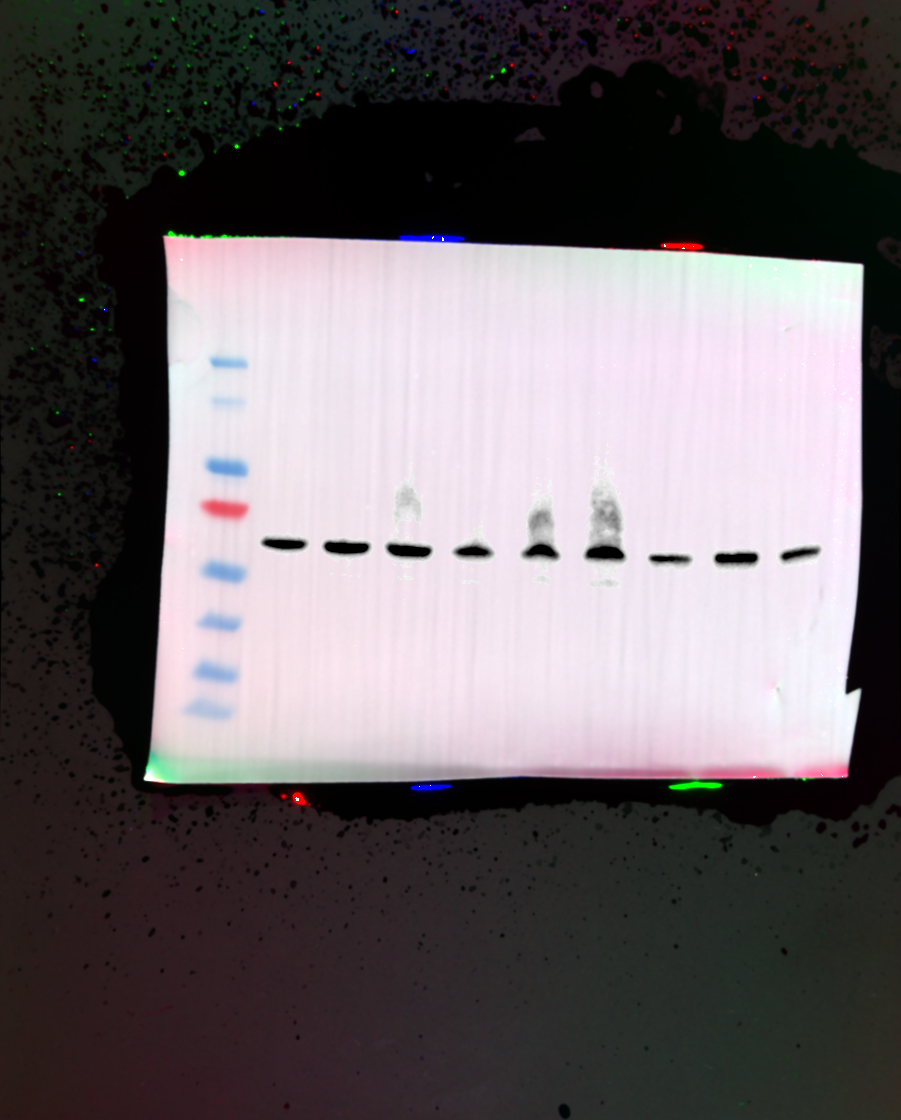

Supplement: Figure 1—figure supplement 1—source data 2. [file elife-85930-fig1-figsupp1-data2.zip › Figure 1 - Figure supplement 1- source data 2/Figure 1 - Figure Supplement 1S/Figure 1 - Figure Supplement 1S representative blots - raw/dnPI3K_Akt_2nd replicate.tif]

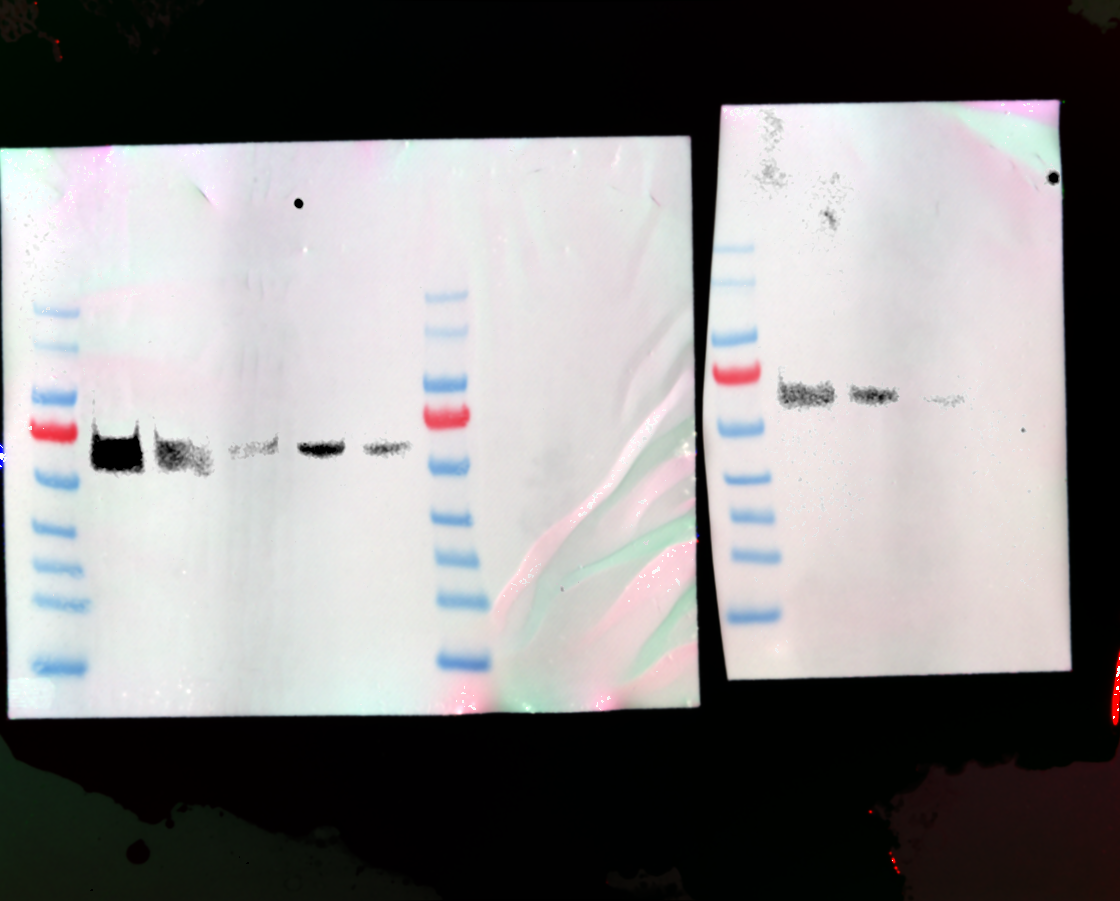

Supplement: Figure 1—figure supplement 1—source data 2. [file elife-85930-fig1-figsupp1-data2.zip › Figure 1 - Figure supplement 1- source data 2/Figure 1 - Figure Supplement 1S/Blots used for Figure 1 - Figure Supplement 1S graph- raw/dnPI3K_pAkt_3rd replicate.tif]

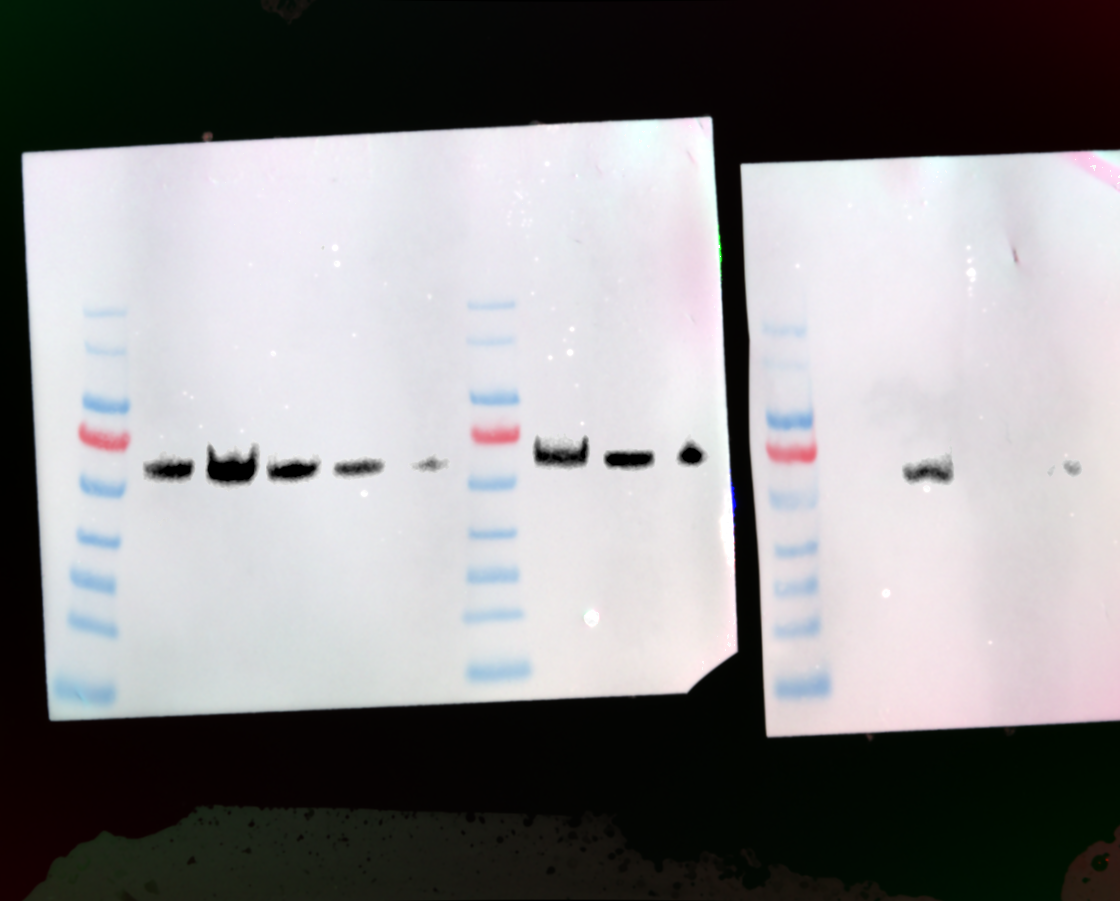

Supplement: Figure 1—figure supplement 1—source data 2. [file elife-85930-fig1-figsupp1-data2.zip › Figure 1 - Figure supplement 1- source data 2/Figure 1 - Figure Supplement 1S/Blots used for Figure 1 - Figure Supplement 1S graph- raw/dnPI3K_pAkt_1st replicate.tif]

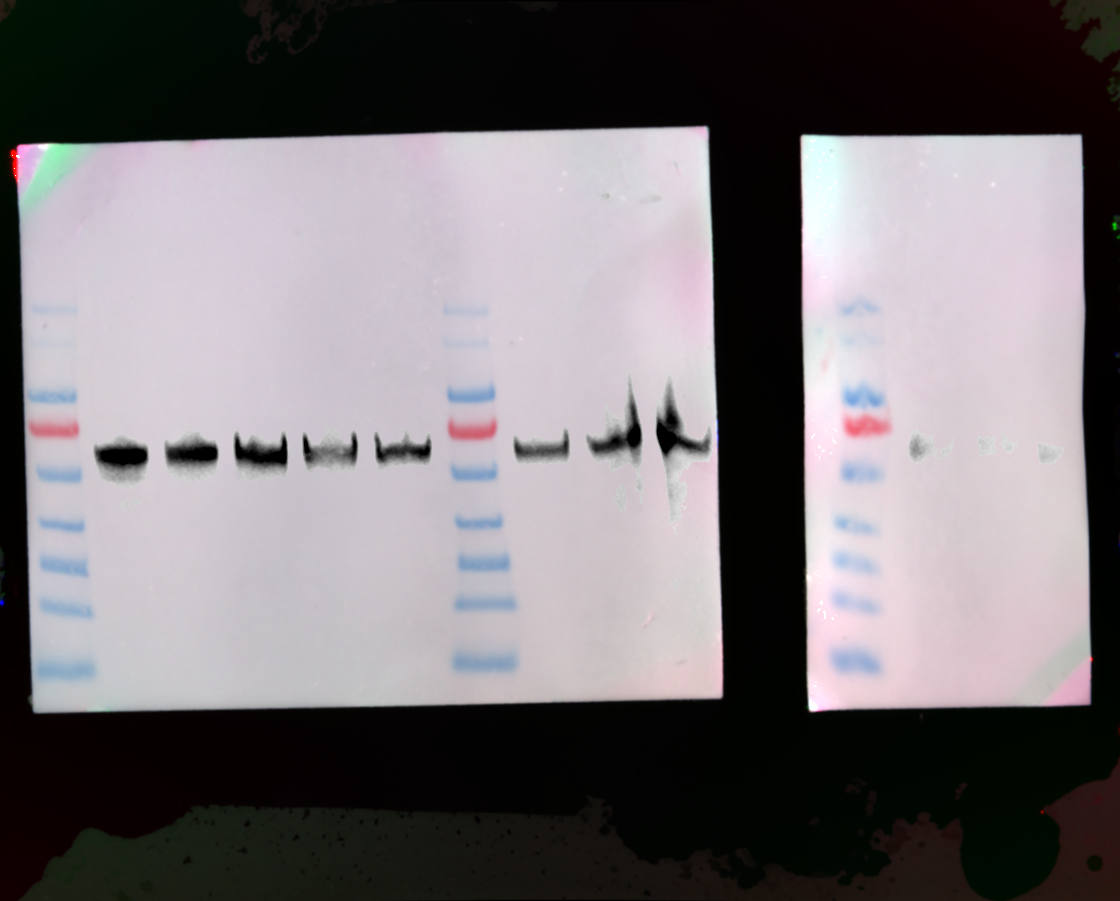

Supplement: Figure 1—figure supplement 1—source data 2. [file elife-85930-fig1-figsupp1-data2.zip › Figure 1 - Figure supplement 1- source data 2/Figure 1 - Figure Supplement 1S/Blots used for Figure 1 - Figure Supplement 1S graph- raw/dnPI3K_Akt_1st replicate.tif]

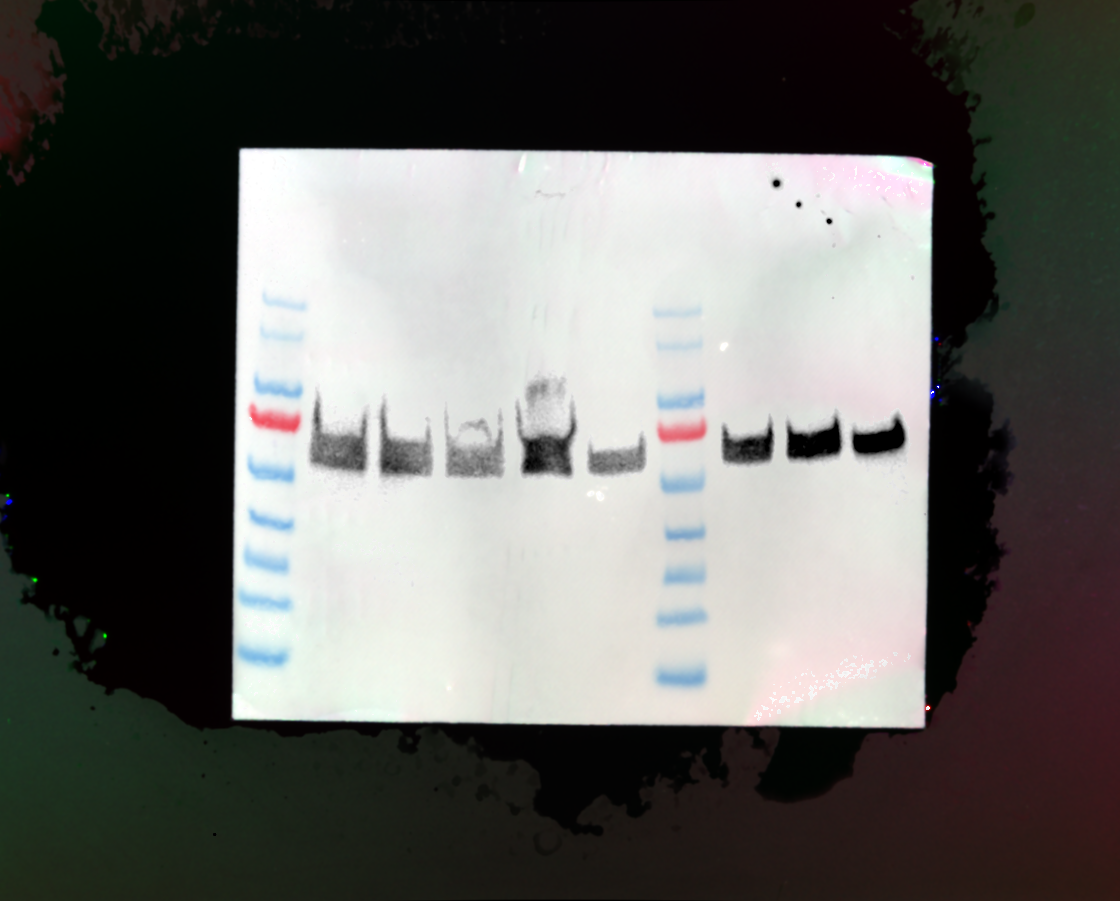

Supplement: Figure 1—figure supplement 1—source data 2. [file elife-85930-fig1-figsupp1-data2.zip › Figure 1 - Figure supplement 1- source data 2/Figure 1 - Figure Supplement 1S/Blots used for Figure 1 - Figure Supplement 1S graph- raw/dnPI3K_Akt_3rd replicate.tif]

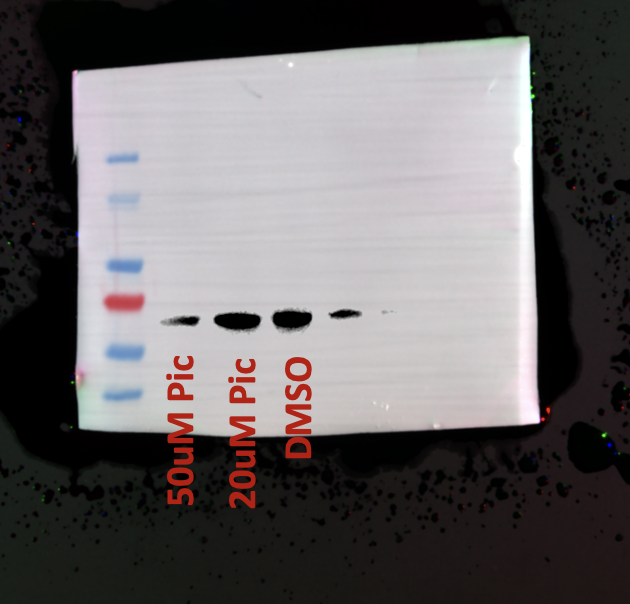

Supplement: Figure 1—figure supplement 1—source data 2. [file elife-85930-fig1-figsupp1-data2.zip › Figure 1 - Figure supplement 1- source data 2/Figure 1 - Figure Supplement 1R/Figure 1 - Figure Supplement 1R representative blots- labelled/Pic_pAkt_2nd replicate_labelled.tiff]

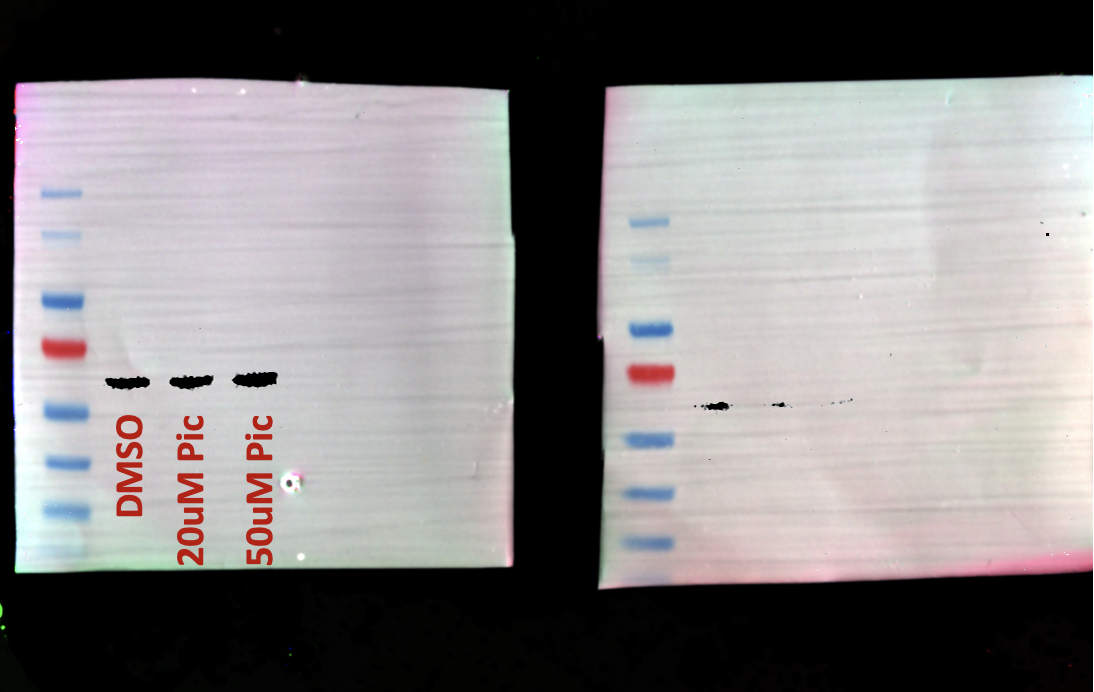

Supplement: Figure 1—figure supplement 1—source data 2. [file elife-85930-fig1-figsupp1-data2.zip › Figure 1 - Figure supplement 1- source data 2/Figure 1 - Figure Supplement 1R/Figure 1 - Figure Supplement 1R representative blots- labelled/Pic_Akt_2nd replicate_labelled.tif]

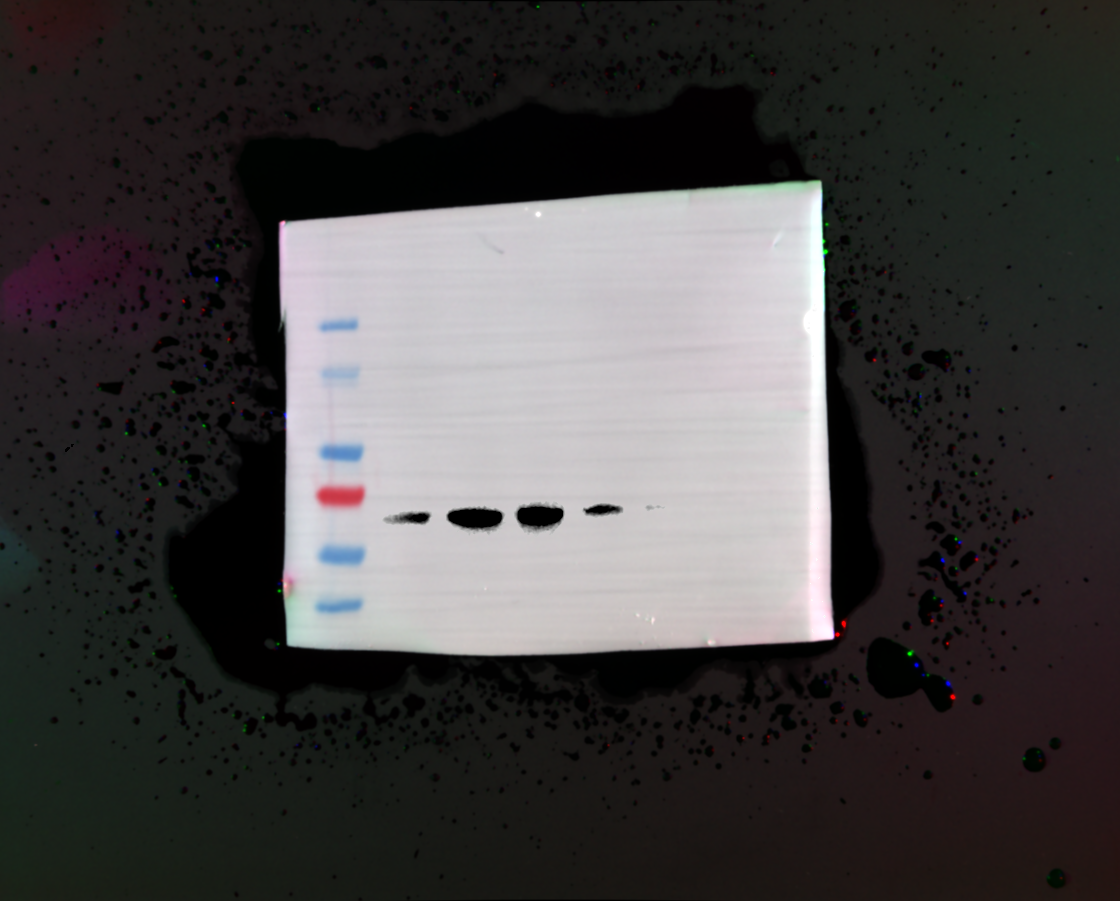

Supplement: Figure 1—figure supplement 1—source data 2. [file elife-85930-fig1-figsupp1-data2.zip › Figure 1 - Figure supplement 1- source data 2/Figure 1 - Figure Supplement 1R/Blots used for Figure 1 - Figure Supplement 1R graph- raw/Pic_pAkt_2nd replicate.tif]

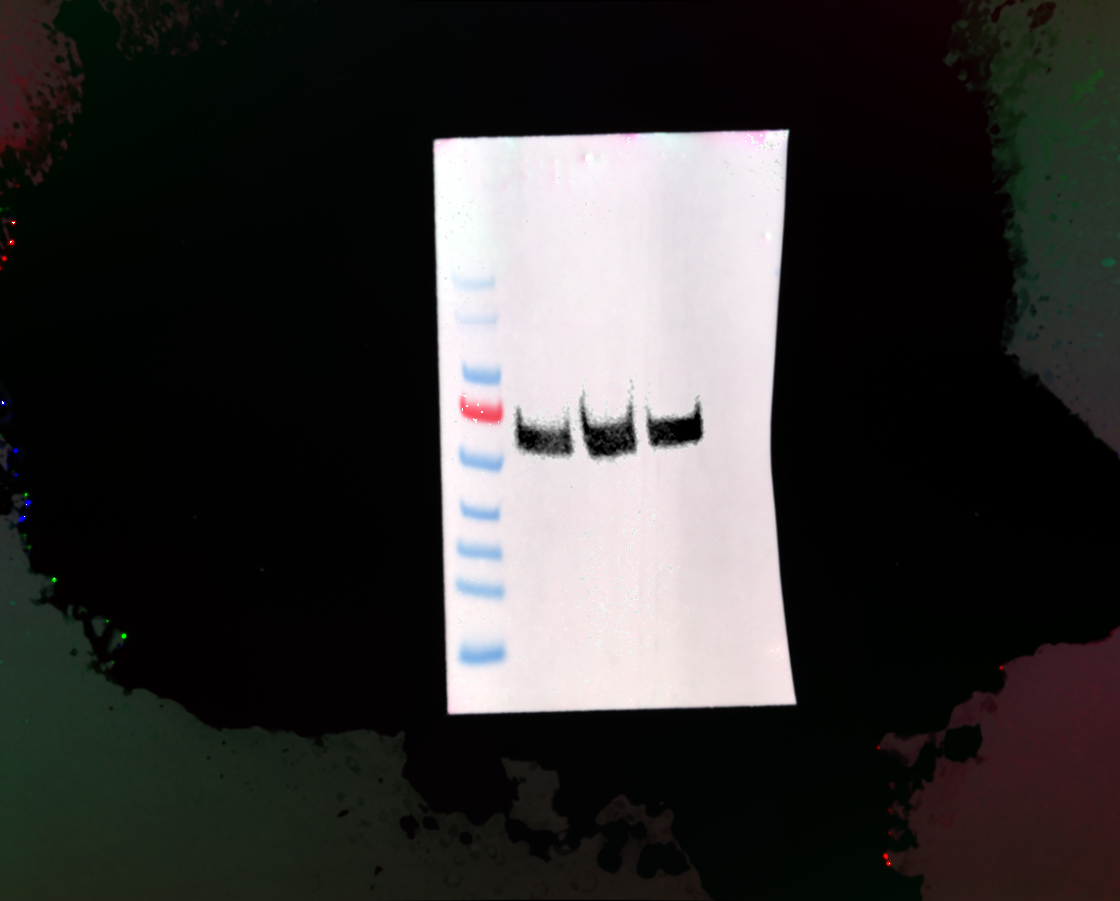

Supplement: Figure 1—figure supplement 1—source data 2. [file elife-85930-fig1-figsupp1-data2.zip › Figure 1 - Figure supplement 1- source data 2/Figure 1 - Figure Supplement 1R/Blots used for Figure 1 - Figure Supplement 1R graph- raw/Pic_Akt_3rd replicate.tif]

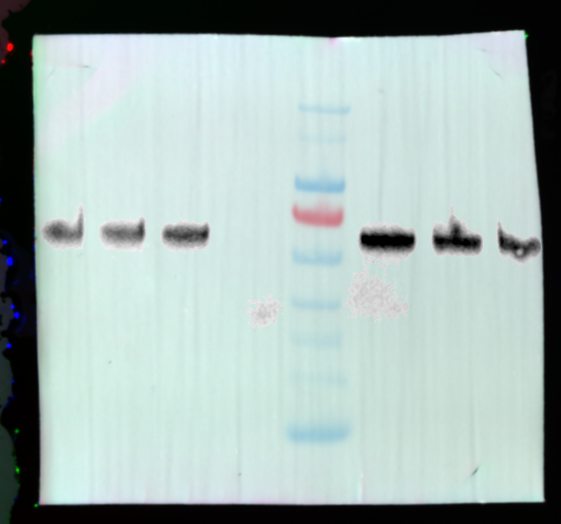

Supplement: Figure 1—figure supplement 1—source data 2. [file elife-85930-fig1-figsupp1-data2.zip › Figure 1 - Figure supplement 1- source data 2/Figure 1 - Figure Supplement 1R/Blots used for Figure 1 - Figure Supplement 1R graph- raw/Pic_Akt_1st replicate.tif]

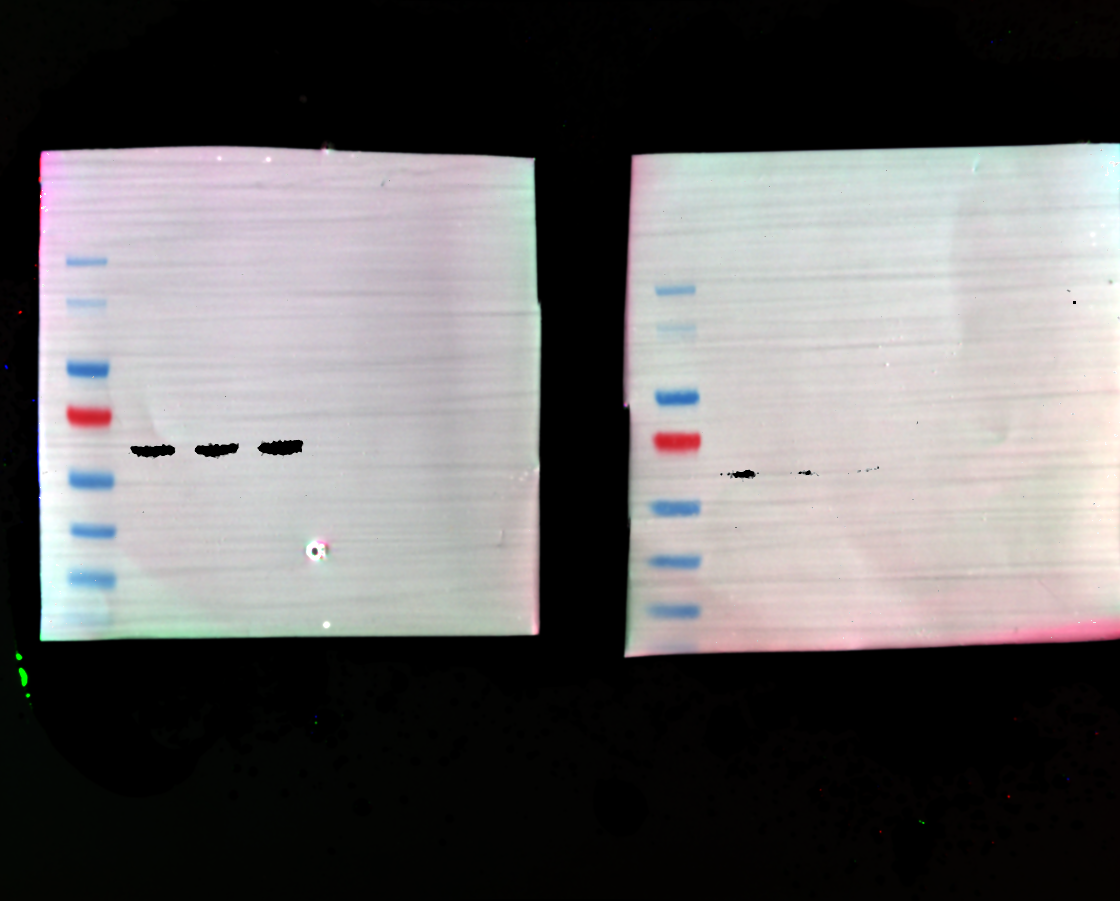

Supplement: Figure 1—figure supplement 1—source data 2. [file elife-85930-fig1-figsupp1-data2.zip › Figure 1 - Figure supplement 1- source data 2/Figure 1 - Figure Supplement 1R/Blots used for Figure 1 - Figure Supplement 1R graph- raw/Pic_Akt_2nd replicate.tif]

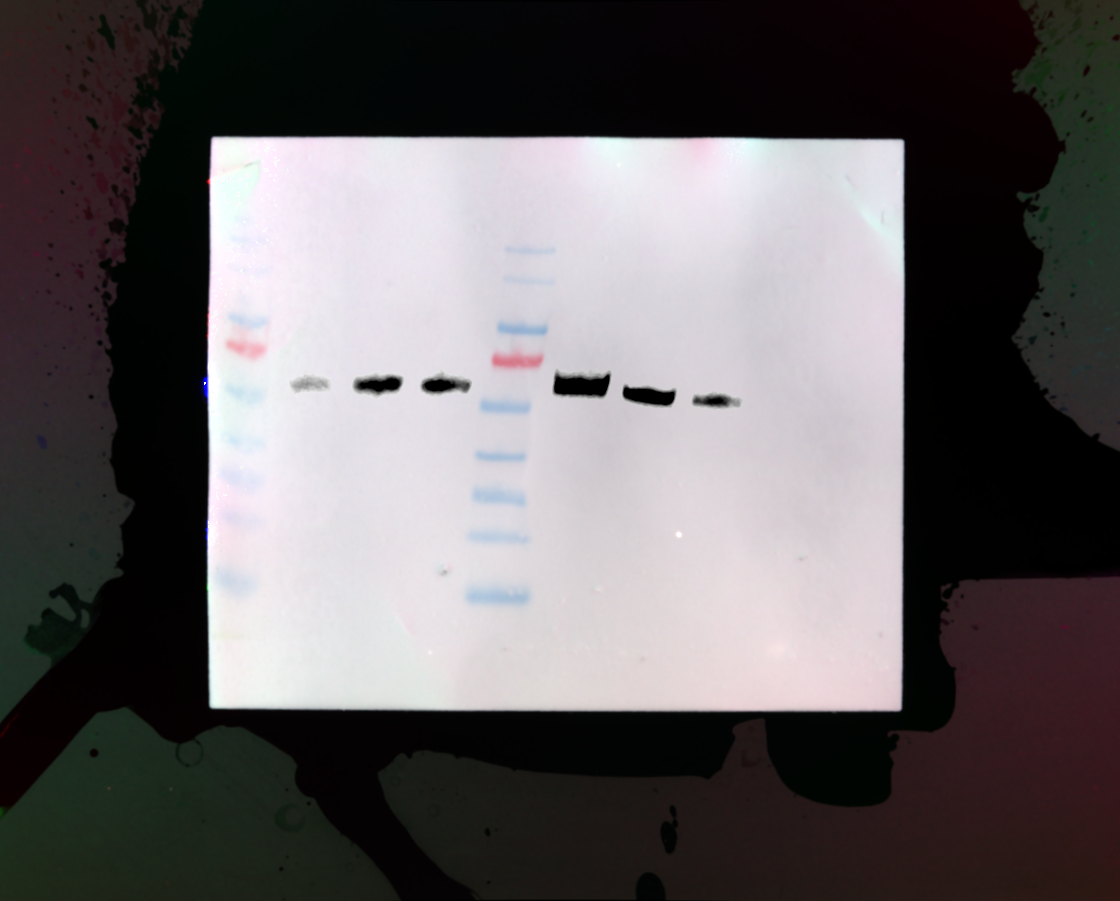

Supplement: Figure 1—figure supplement 1—source data 2. [file elife-85930-fig1-figsupp1-data2.zip › Figure 1 - Figure supplement 1- source data 2/Figure 1 - Figure Supplement 1R/Blots used for Figure 1 - Figure Supplement 1R graph- raw/Pic_pAkt_1st replicate.tif]

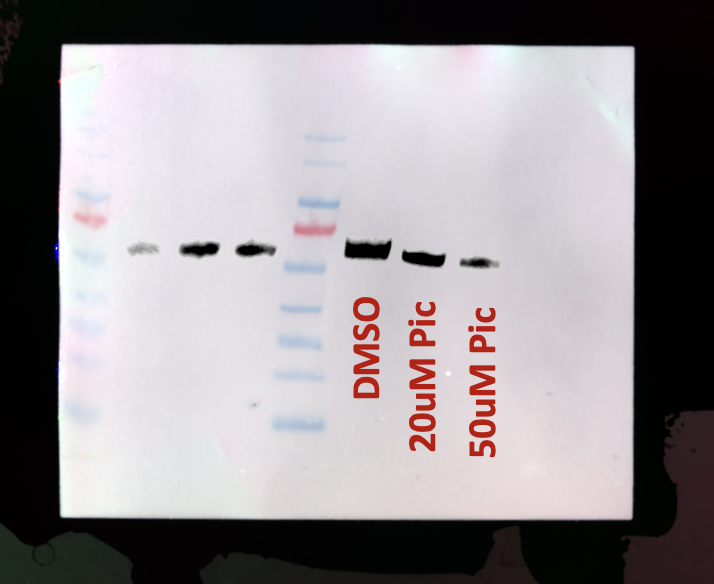

Supplement: Figure 1—figure supplement 1—source data 2. [file elife-85930-fig1-figsupp1-data2.zip › Figure 1 - Figure supplement 1- source data 2/Figure 1 - Figure Supplement 1R/Blots used for Figure 1 - Figure Supplement 1R graph- labelled/Pic_pAkt_1st replicate_labelled.tiff]

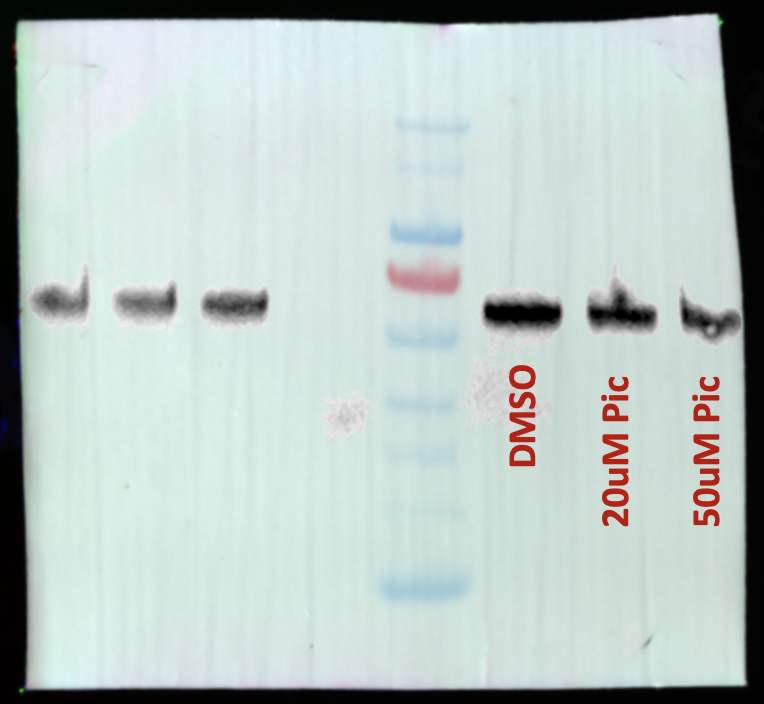

Supplement: Figure 1—figure supplement 1—source data 2. [file elife-85930-fig1-figsupp1-data2.zip › Figure 1 - Figure supplement 1- source data 2/Figure 1 - Figure Supplement 1R/Blots used for Figure 1 - Figure Supplement 1R graph- labelled/Pic_Akt_1st replicate_labelled.tiff]

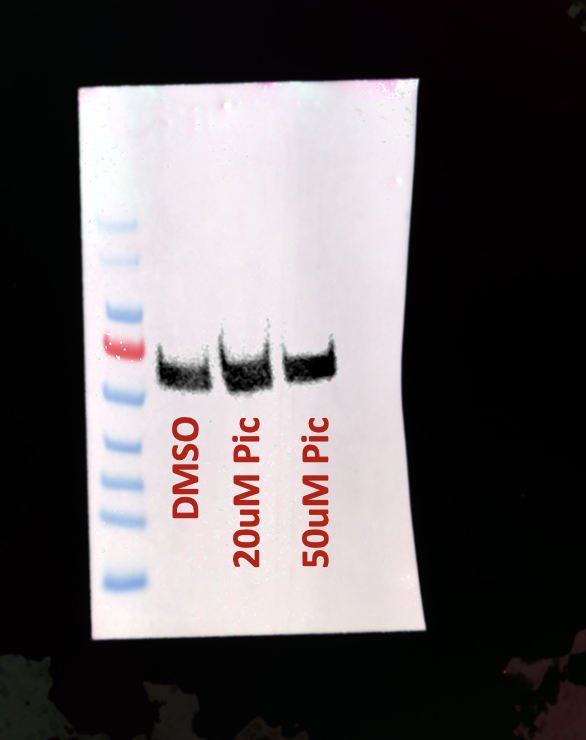

Supplement: Figure 1—figure supplement 1—source data 2. [file elife-85930-fig1-figsupp1-data2.zip › Figure 1 - Figure supplement 1- source data 2/Figure 1 - Figure Supplement 1R/Blots used for Figure 1 - Figure Supplement 1R graph- labelled/Pic_Akt_3rd replicate_labelled.tif]

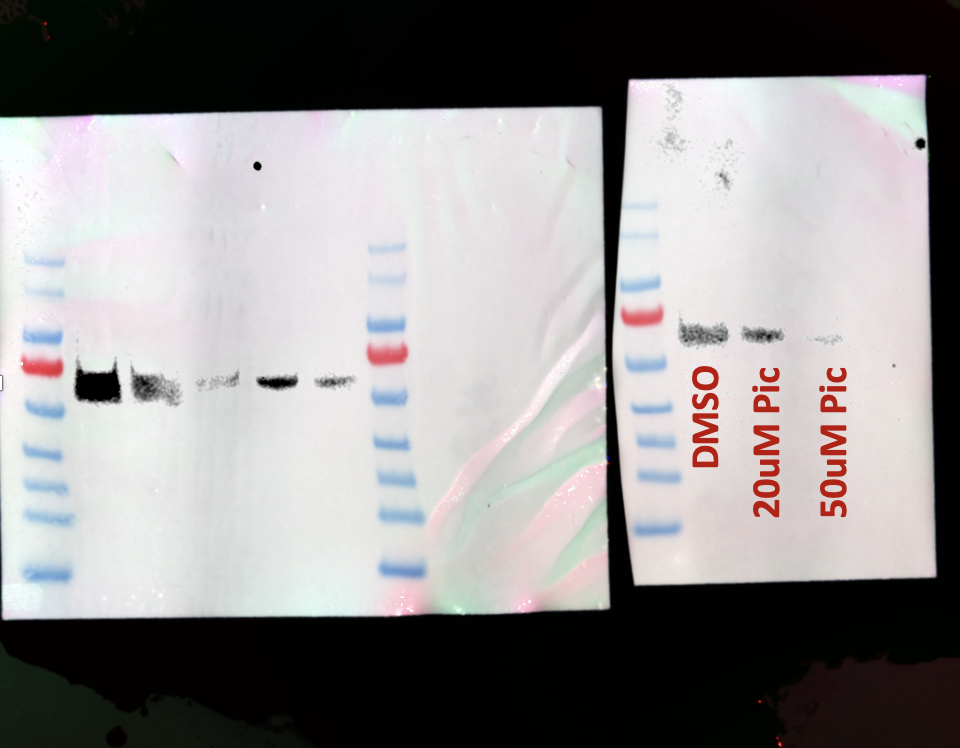

Supplement: Figure 1—figure supplement 1—source data 2. [file elife-85930-fig1-figsupp1-data2.zip › Figure 1 - Figure supplement 1- source data 2/Figure 1 - Figure Supplement 1R/Blots used for Figure 1 - Figure Supplement 1R graph- labelled/Pic_pAkt_3rd replicate_labelled.tif]

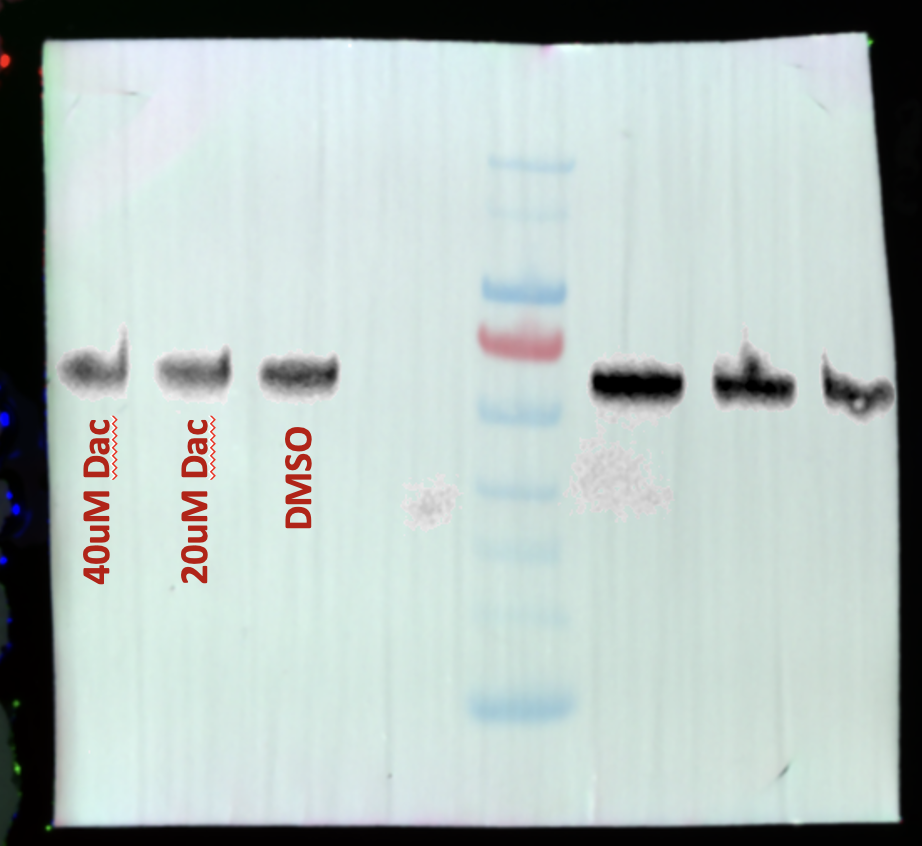

Supplement: Figure 1—figure supplement 1—source data 2. [file elife-85930-fig1-figsupp1-data2.zip › Figure 1 - Figure supplement 1- source data 2/Figure 1 - Figure Supplement 1Q/Blots used for Figure 1 - Figure Supplement 1Q graph- labelled/Dac_Akt_1st replicate_labelled.tiff]

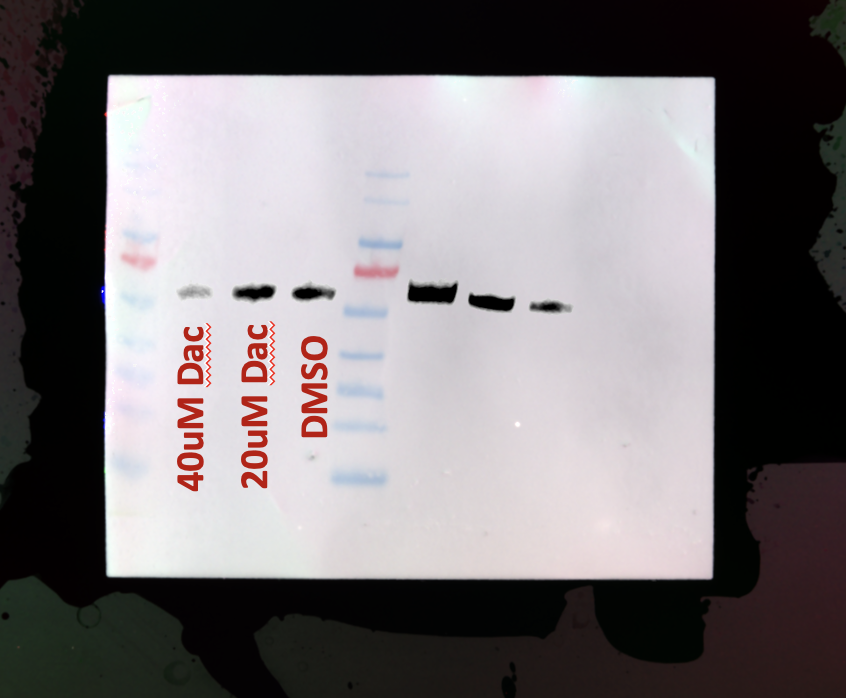

Supplement: Figure 1—figure supplement 1—source data 2. [file elife-85930-fig1-figsupp1-data2.zip › Figure 1 - Figure supplement 1- source data 2/Figure 1 - Figure Supplement 1Q/Blots used for Figure 1 - Figure Supplement 1Q graph- labelled/Dac_pAkt_1st replicate_labelled.tiff]

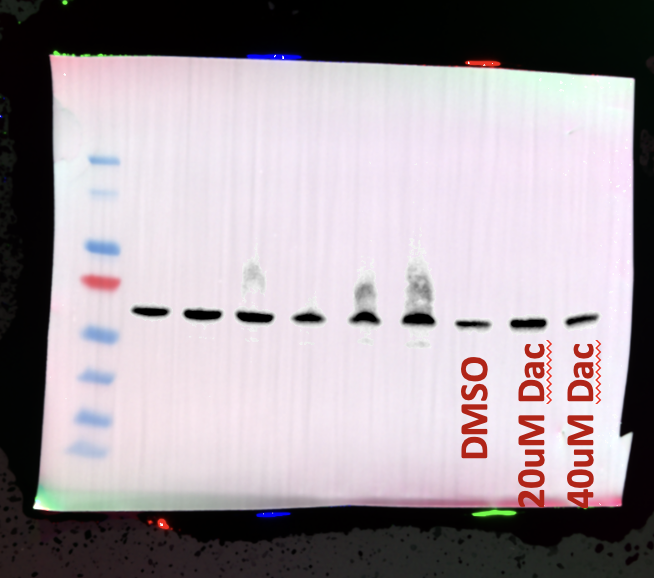

Supplement: Figure 1—figure supplement 1—source data 2. [file elife-85930-fig1-figsupp1-data2.zip › Figure 1 - Figure supplement 1- source data 2/Figure 1 - Figure Supplement 1Q/Blots used for Figure 1 - Figure Supplement 1Q graph- labelled/Dac_Akt_2nd replicate_labelled.tiff]

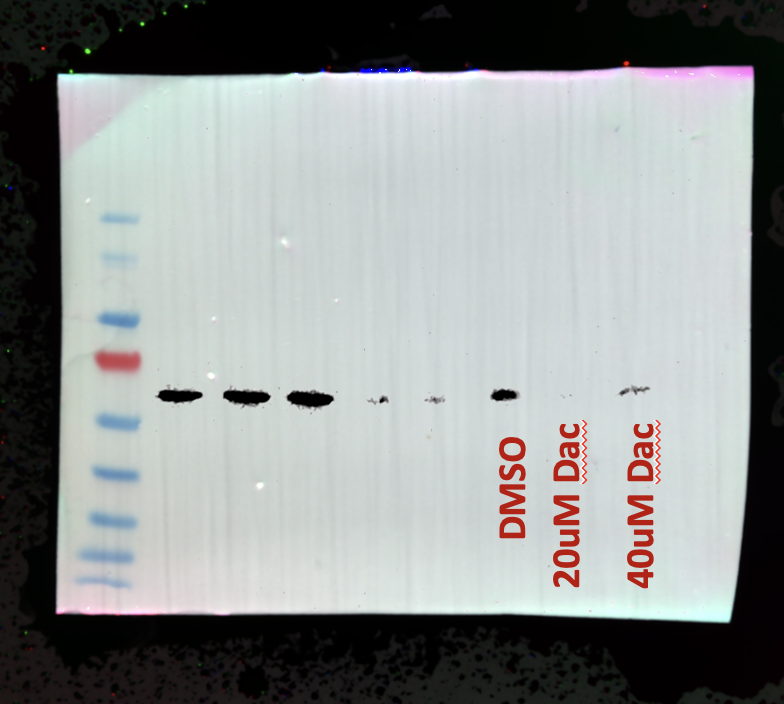

Supplement: Figure 1—figure supplement 1—source data 2. [file elife-85930-fig1-figsupp1-data2.zip › Figure 1 - Figure supplement 1- source data 2/Figure 1 - Figure Supplement 1Q/Blots used for Figure 1 - Figure Supplement 1Q graph- labelled/Dac_pAkt_2nd replicate_labelled.tiff]

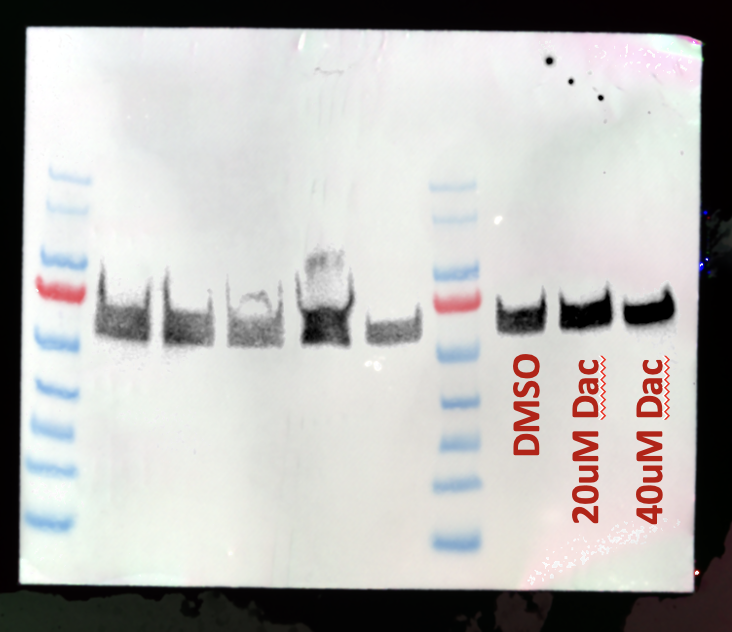

Supplement: Figure 1—figure supplement 1—source data 2. [file elife-85930-fig1-figsupp1-data2.zip › Figure 1 - Figure supplement 1- source data 2/Figure 1 - Figure Supplement 1Q/Blots used for Figure 1 - Figure Supplement 1Q graph- labelled/Dac_Akt_3rd replicate_labelled.tiff]

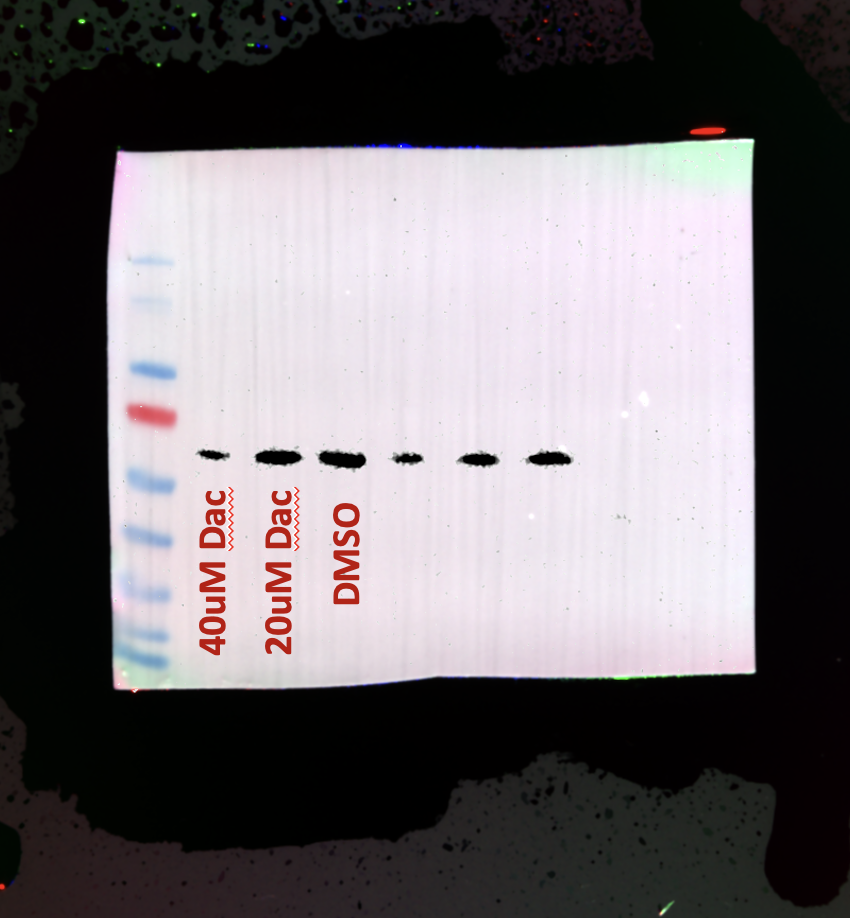

Supplement: Figure 1—figure supplement 1—source data 2. [file elife-85930-fig1-figsupp1-data2.zip › Figure 1 - Figure supplement 1- source data 2/Figure 1 - Figure Supplement 1Q/Blots used for Figure 1 - Figure Supplement 1Q graph- labelled/Dac_pAkt_3rd replicate_labelled.tiff]

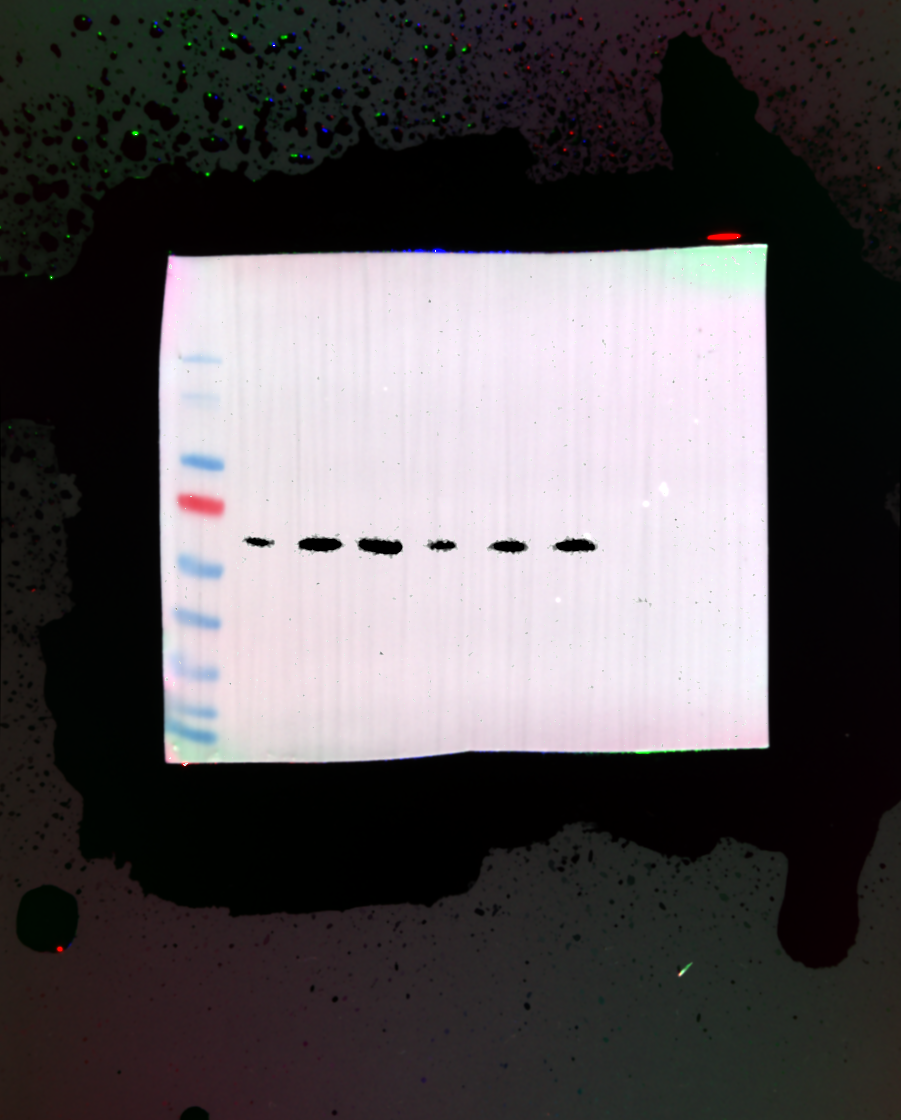

Supplement: Figure 1—figure supplement 1—source data 2. [file elife-85930-fig1-figsupp1-data2.zip › Figure 1 - Figure supplement 1- source data 2/Figure 1 - Figure Supplement 1Q/Figure 1 - Figure Supplement 1Q representative blots - raw/Dac_pAkt_3rd replicate.tif]

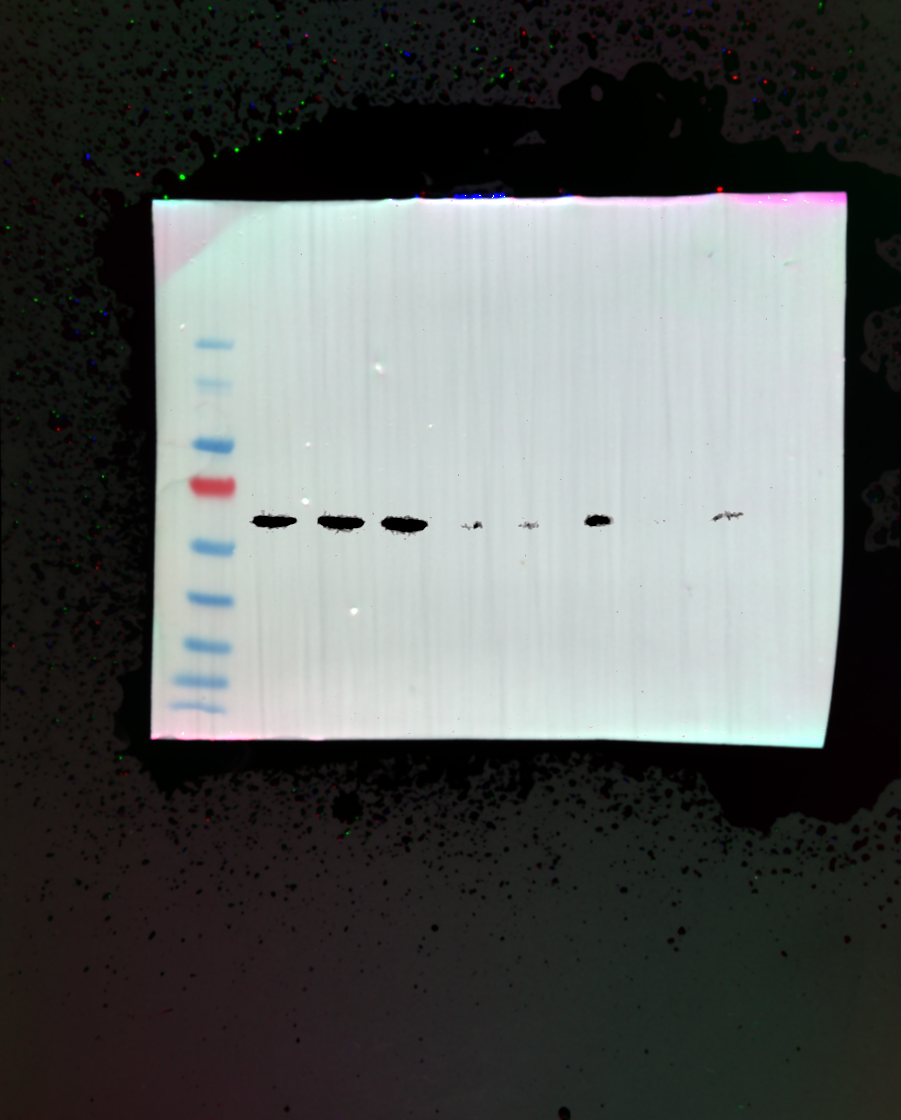

Supplement: Figure 1—figure supplement 1—source data 2. [file elife-85930-fig1-figsupp1-data2.zip › Figure 1 - Figure supplement 1- source data 2/Figure 1 - Figure Supplement 1Q/Blots used for Figure 1 - Figure Supplement 1Q graph- raw/Dac_pAkt_2nd replicate.tiff]

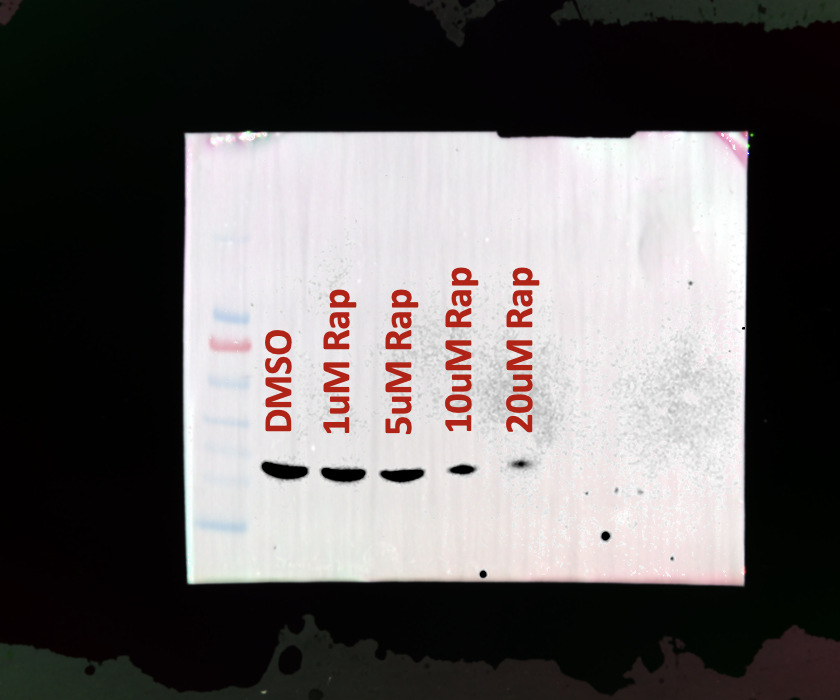

Supplement: Figure 1—figure supplement 3—source data 2. [file elife-85930-fig1-figsupp3-data2.zip › Figure 1 - Figure supplement 3- source data 2/Figure 1 - Figure Supplement 3G representative blots - labelled/Rapamycin_pS6_2nd replicate.tiff]

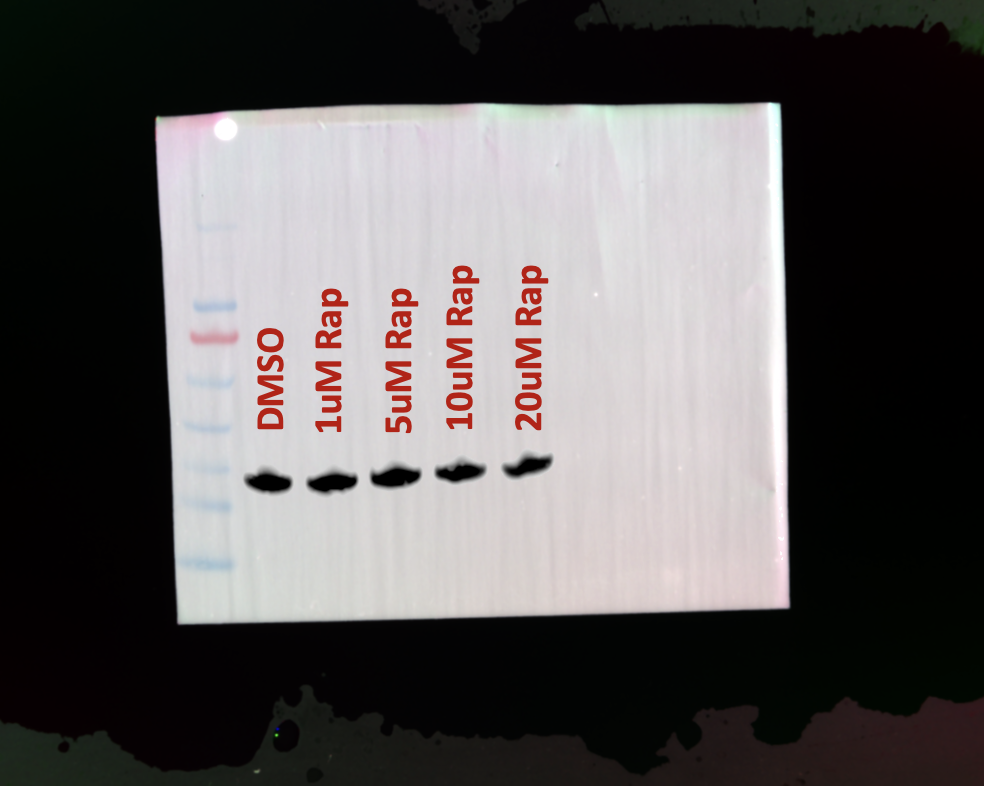

Supplement: Figure 1—figure supplement 3—source data 2. [file elife-85930-fig1-figsupp3-data2.zip › Figure 1 - Figure supplement 3- source data 2/Figure 1 - Figure Supplement 3G representative blots - labelled/Rapamycin_S6_2nd replicate_labelled.tiff]

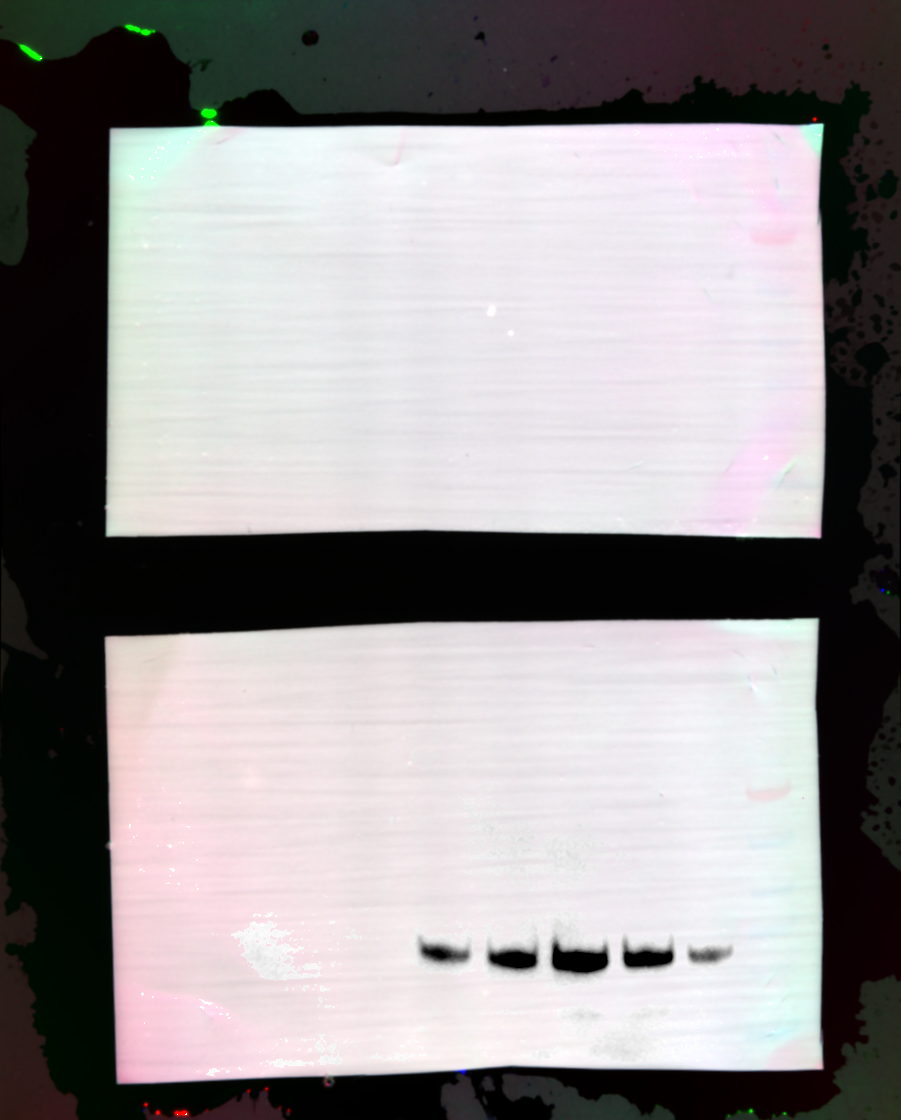

Supplement: Figure 1—figure supplement 3—source data 2. [file elife-85930-fig1-figsupp3-data2.zip › Figure 1 - Figure supplement 3- source data 2/Blots used for Figure 1 - Figure Supplement 3G graph- raw/Rapamycin_S6_1st replicate.tif]

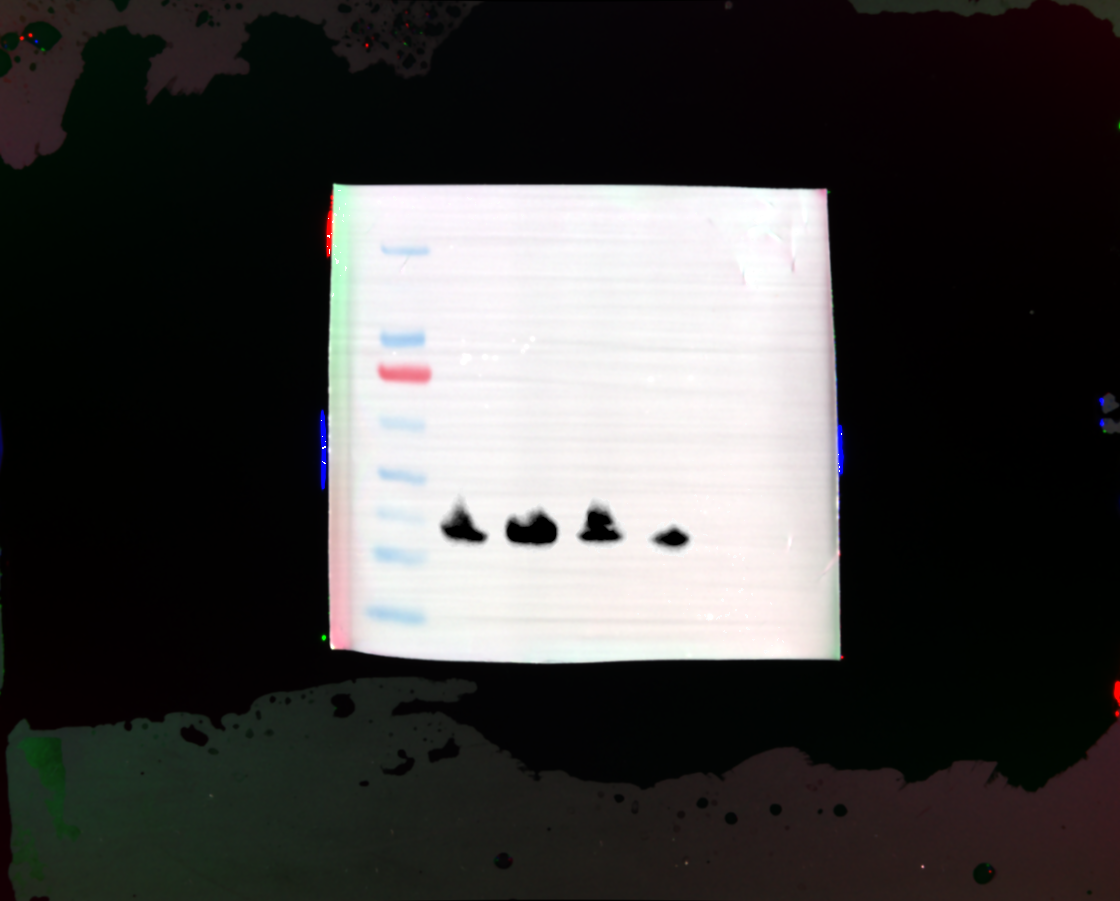

Supplement: Figure 1—figure supplement 3—source data 2. [file elife-85930-fig1-figsupp3-data2.zip › Figure 1 - Figure supplement 3- source data 2/Blots used for Figure 1 - Figure Supplement 3G graph- raw/Rapamycin_pS6_1st replicate.tif]

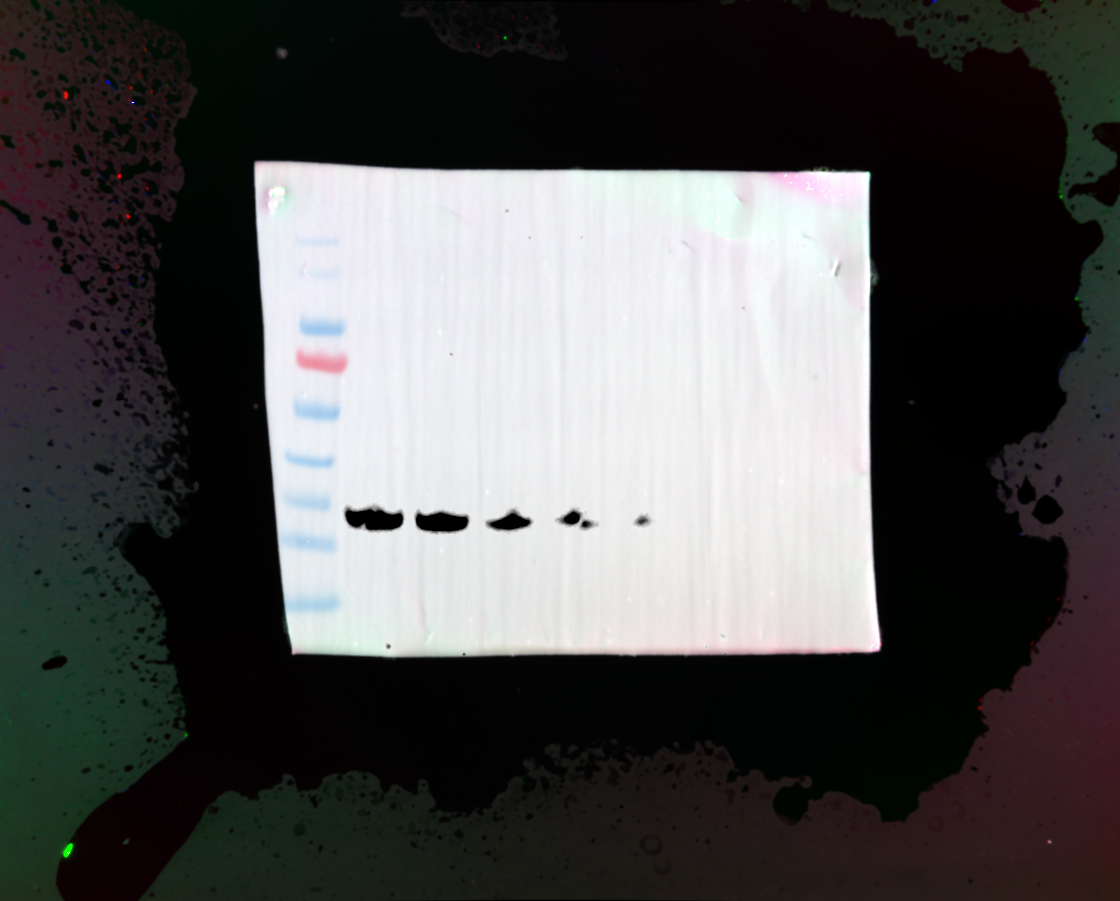

Supplement: Figure 1—figure supplement 3—source data 2. [file elife-85930-fig1-figsupp3-data2.zip › Figure 1 - Figure supplement 3- source data 2/Blots used for Figure 1 - Figure Supplement 3G graph- raw/Rapamycin_pS6_3rd replicate.tif]

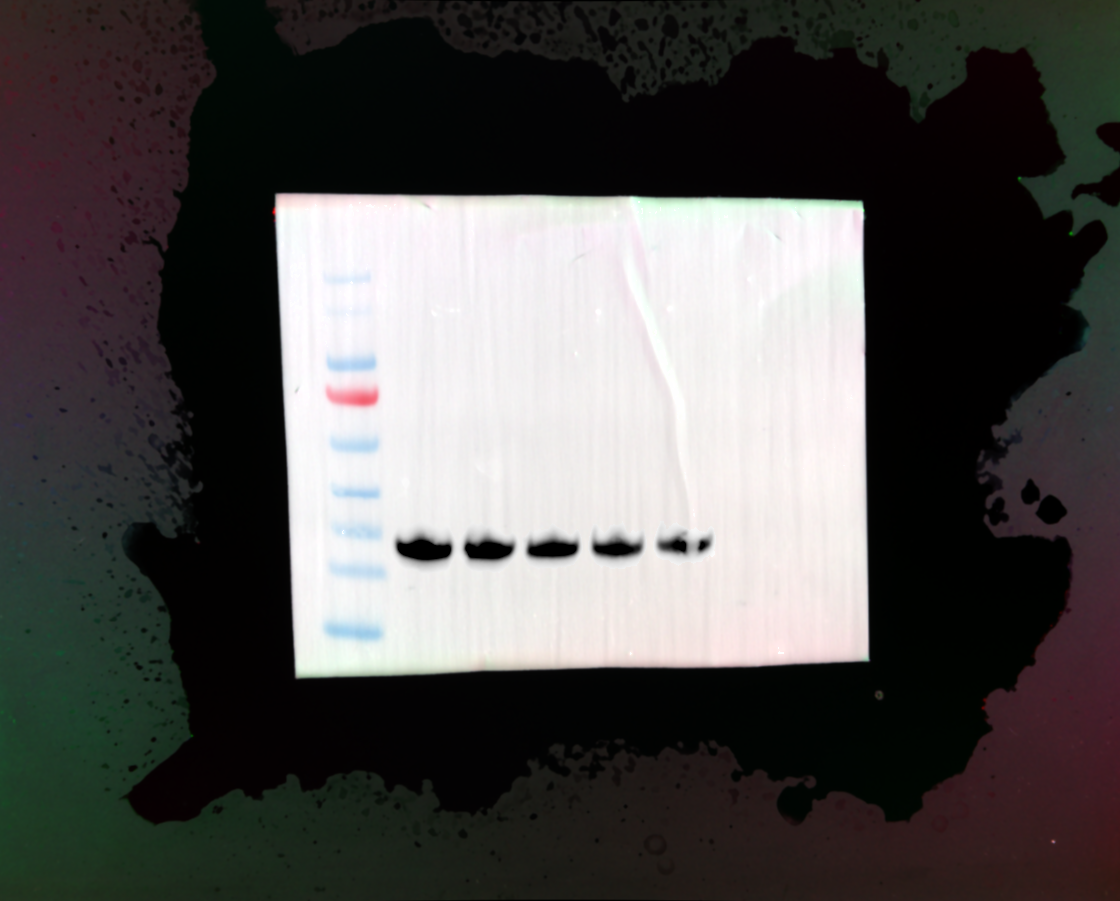

Supplement: Figure 1—figure supplement 3—source data 2. [file elife-85930-fig1-figsupp3-data2.zip › Figure 1 - Figure supplement 3- source data 2/Blots used for Figure 1 - Figure Supplement 3G graph- raw/Rapamycin_S6_3rd replicate.tif]

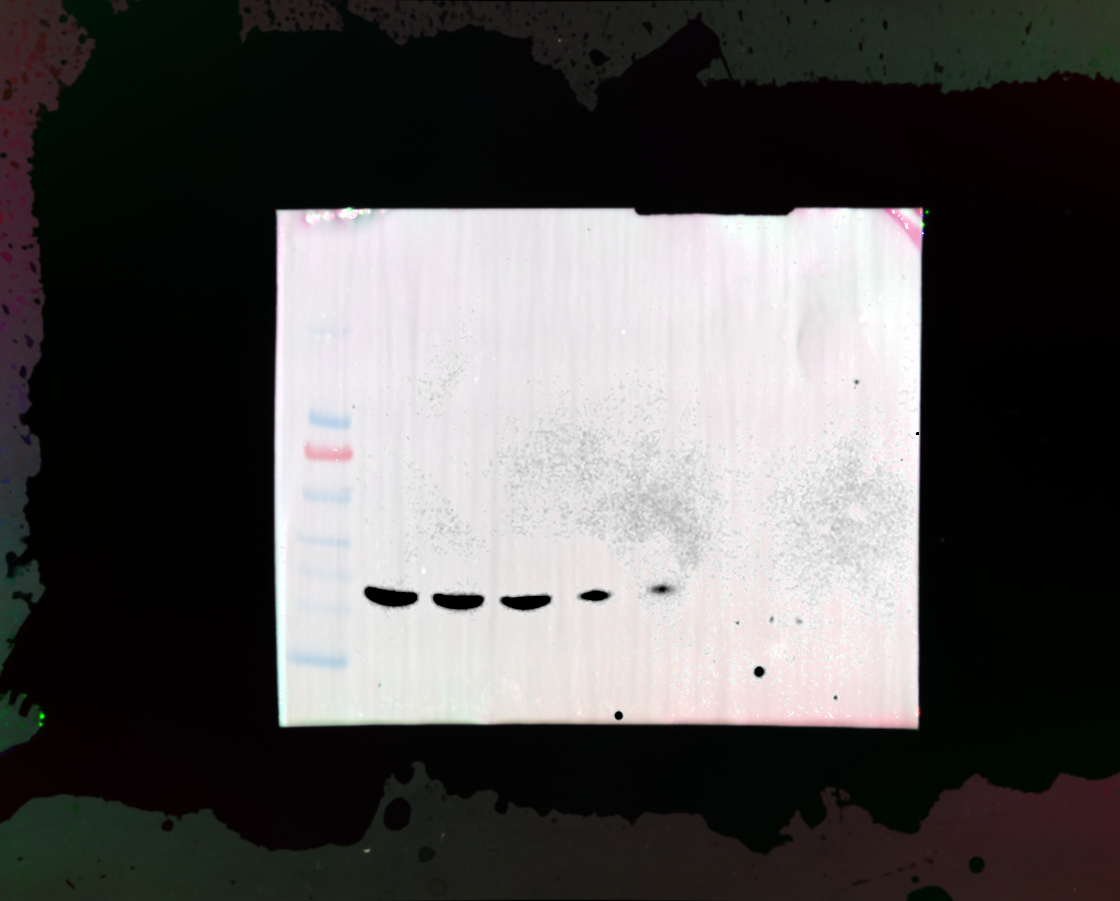

Supplement: Figure 1—figure supplement 3—source data 2. [file elife-85930-fig1-figsupp3-data2.zip › Figure 1 - Figure supplement 3- source data 2/Blots used for Figure 1 - Figure Supplement 3G graph- raw/Rapamycin_pS6_2nd replicate.tif]

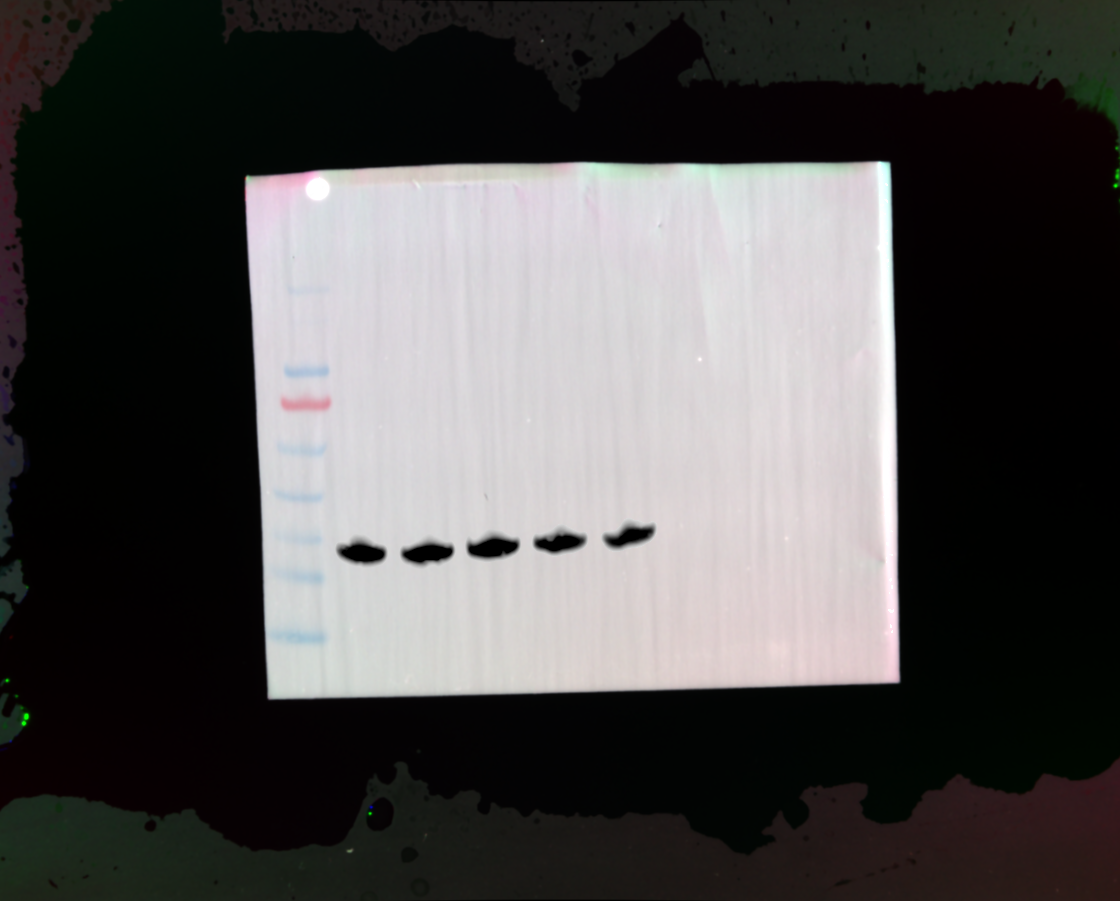

Supplement: Figure 1—figure supplement 3—source data 2. [file elife-85930-fig1-figsupp3-data2.zip › Figure 1 - Figure supplement 3- source data 2/Blots used for Figure 1 - Figure Supplement 3G graph- raw/Rapamycin_S6_2nd replicate.tif]

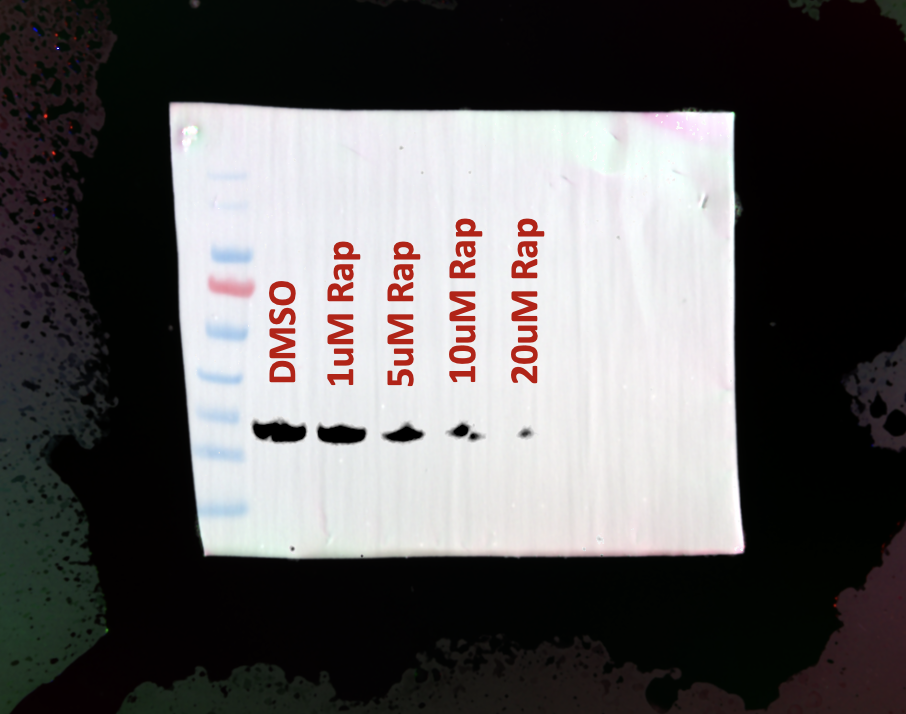

Supplement: Figure 1—figure supplement 3—source data 2. [file elife-85930-fig1-figsupp3-data2.zip › Figure 1 - Figure supplement 3- source data 2/Blots used for Figure 1 - Figure Supplement 3G graph- labelled/Rapamycin_pS6_3rd replicate.tiff]

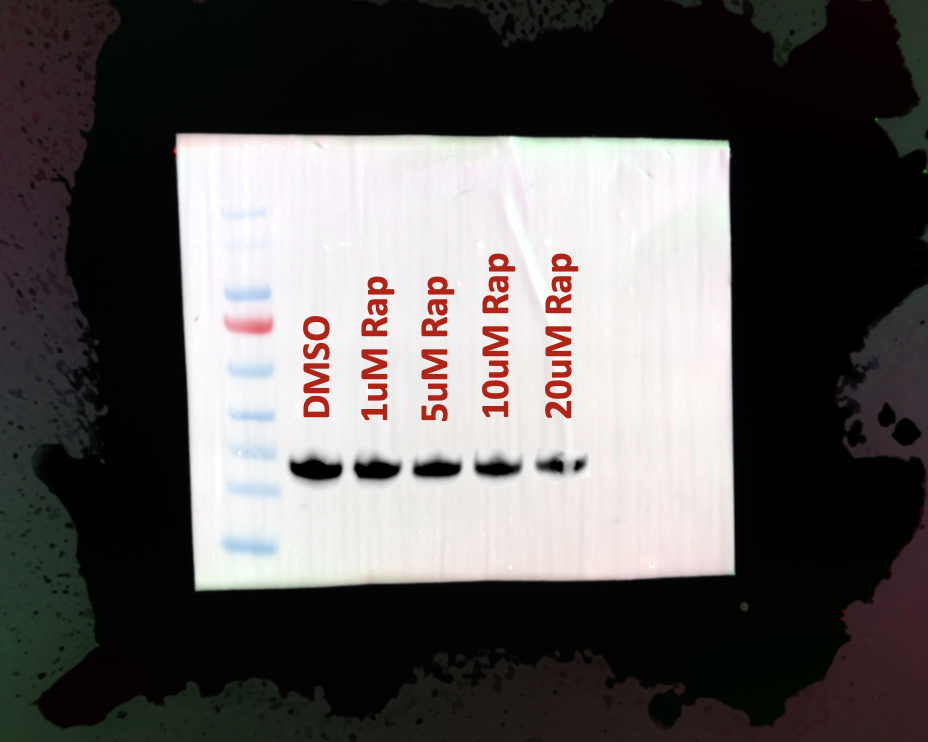

Supplement: Figure 1—figure supplement 3—source data 2. [file elife-85930-fig1-figsupp3-data2.zip › Figure 1 - Figure supplement 3- source data 2/Blots used for Figure 1 - Figure Supplement 3G graph- labelled/Rapamycin_S6_3rd replicate.tiff]

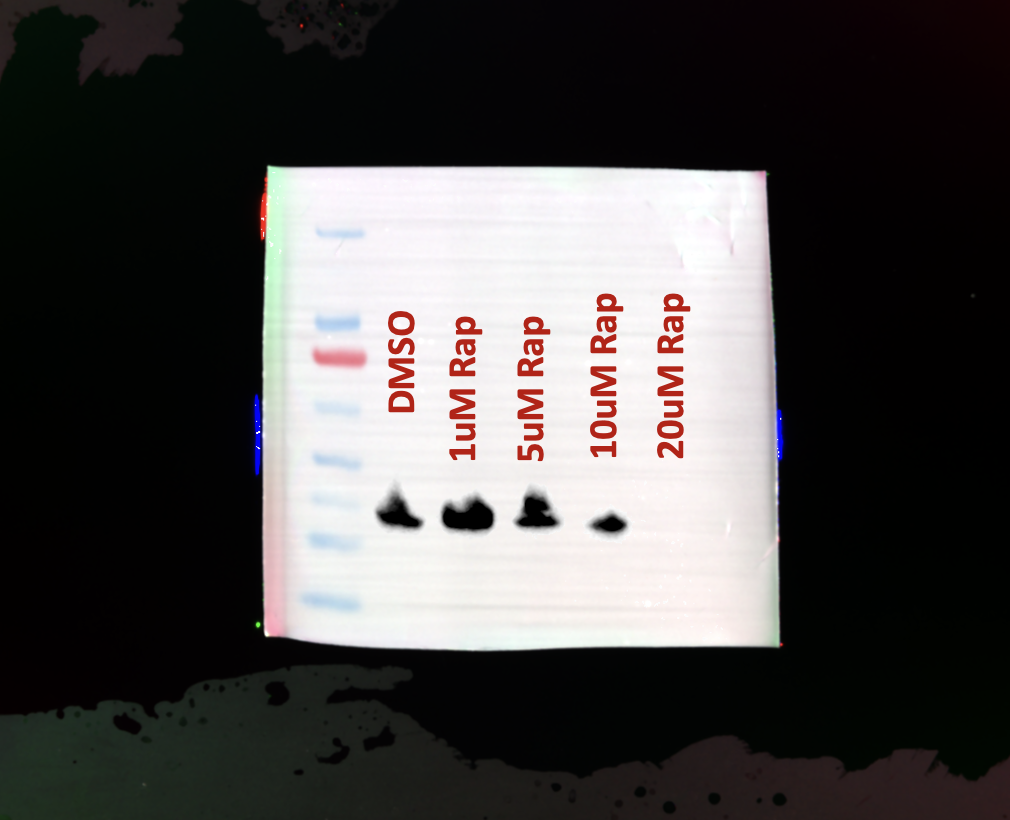

Supplement: Figure 1—figure supplement 3—source data 2. [file elife-85930-fig1-figsupp3-data2.zip › Figure 1 - Figure supplement 3- source data 2/Blots used for Figure 1 - Figure Supplement 3G graph- labelled/Rapamycin_pS6_1st replicate_labelled.tiff]

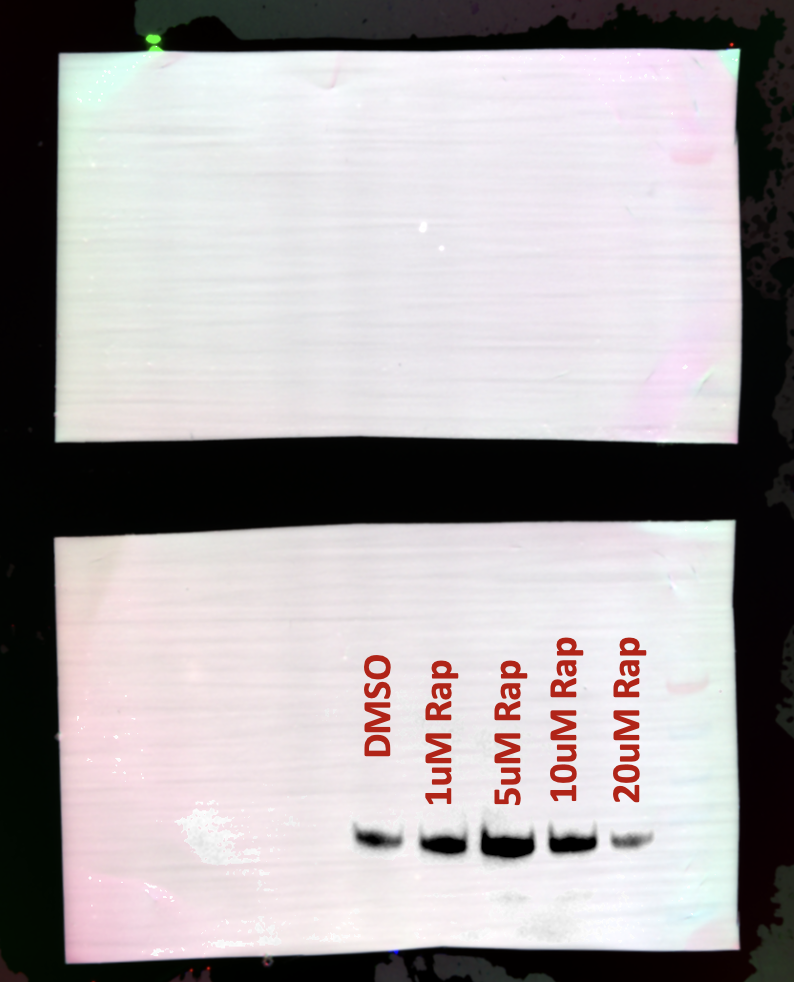

Supplement: Figure 1—figure supplement 3—source data 2. [file elife-85930-fig1-figsupp3-data2.zip › Figure 1 - Figure supplement 3- source data 2/Blots used for Figure 1 - Figure Supplement 3G graph- labelled/Rapamycin_S6_1st replicate_labelled.tiff]

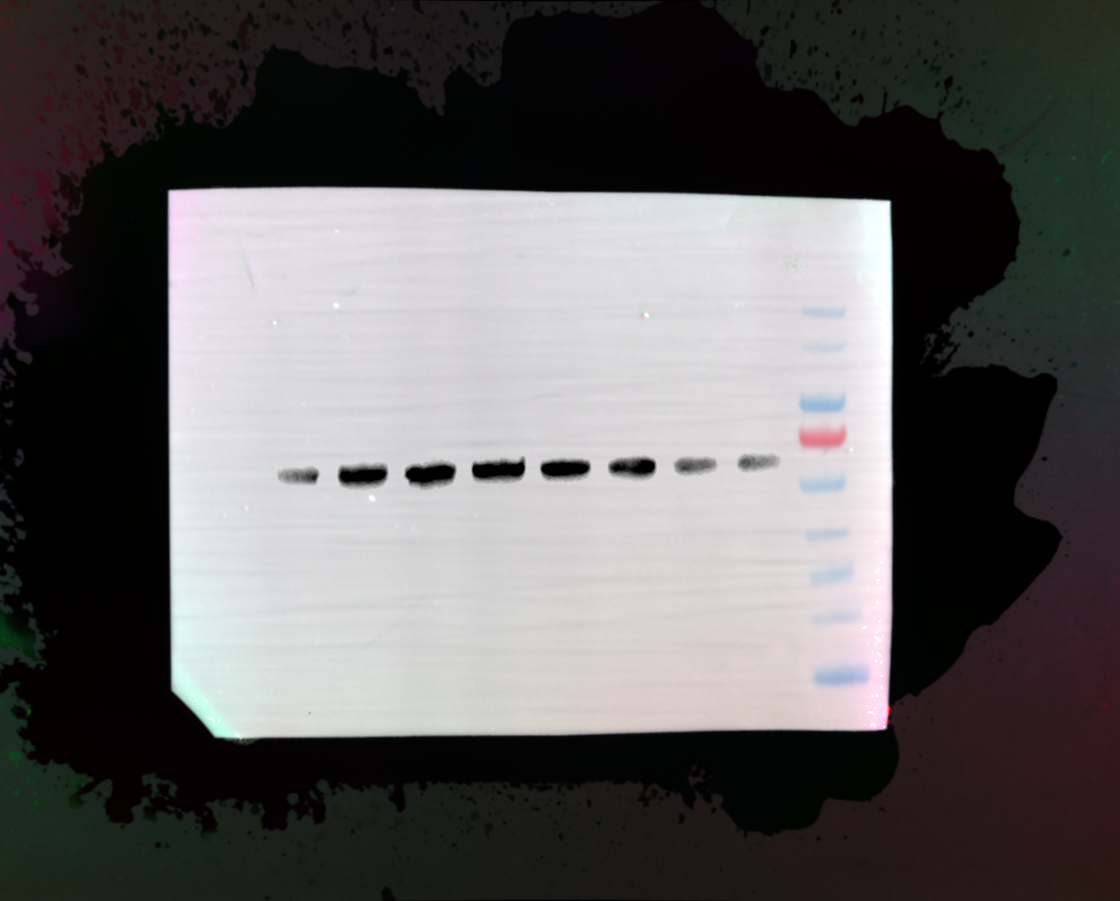

Supplement: Figure 1—figure supplement 6—source data 2. [file elife-85930-fig1-figsupp6-data2.zip › Figure 1 - Figure supplement 6- source data 2/Blots used for Figure 1 - Figure Supplement 6H graph- raw/VOOH_Akt_1st replicate.tif]

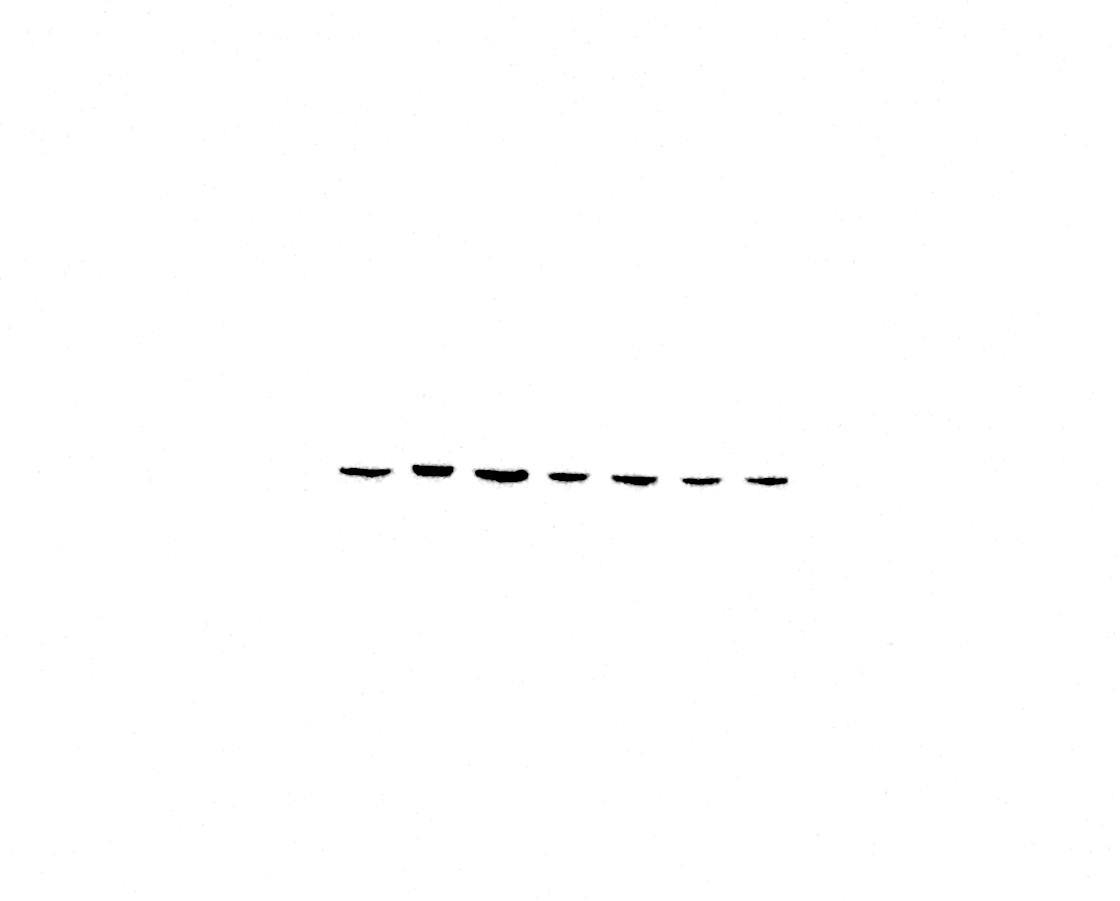

Supplement: Figure 1—figure supplement 6—source data 2. [file elife-85930-fig1-figsupp6-data2.zip › Figure 1 - Figure supplement 6- source data 2/Blots used for Figure 1 - Figure Supplement 6H graph- raw/VOOH_Akt_3rd replicate.tif]

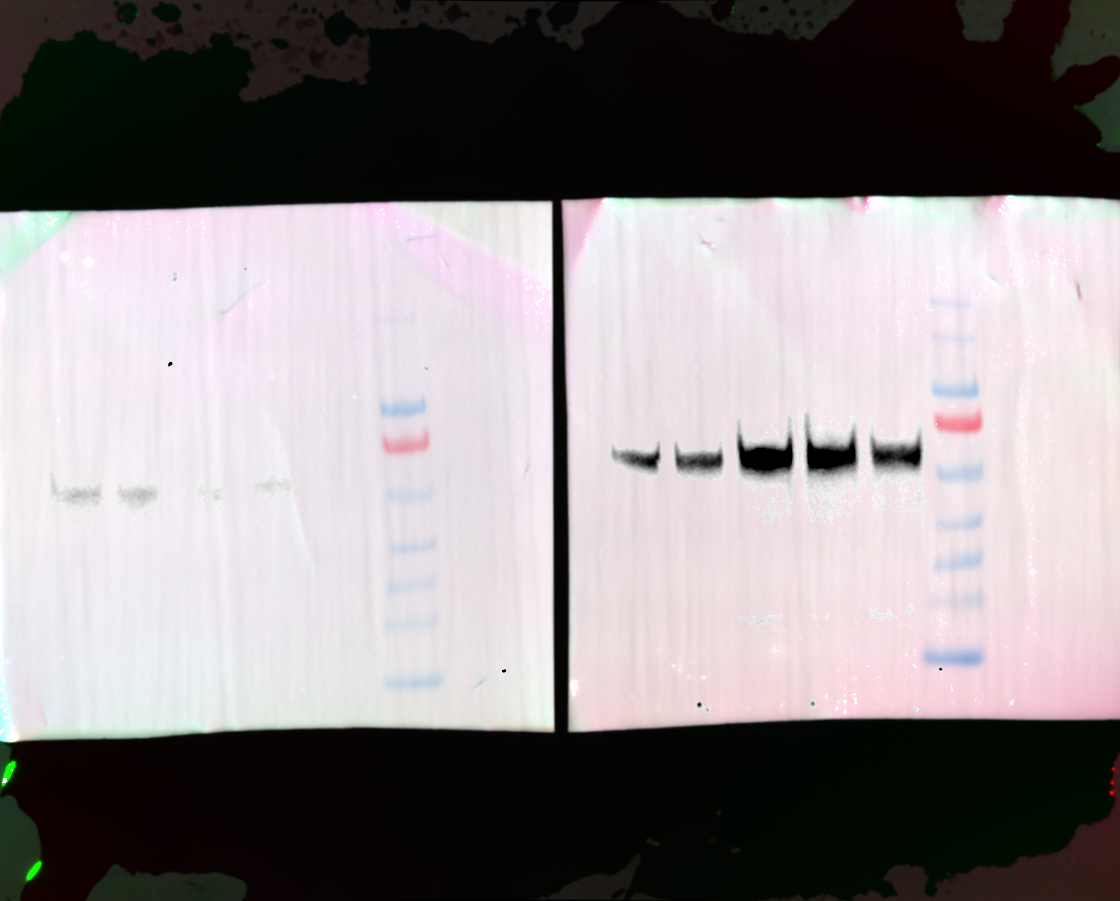

Supplement: Figure 1—figure supplement 6—source data 2. [file elife-85930-fig1-figsupp6-data2.zip › Figure 1 - Figure supplement 6- source data 2/Blots used for Figure 1 - Figure Supplement 6H graph- raw/VOOH_pAkt_1st replicate.tif]

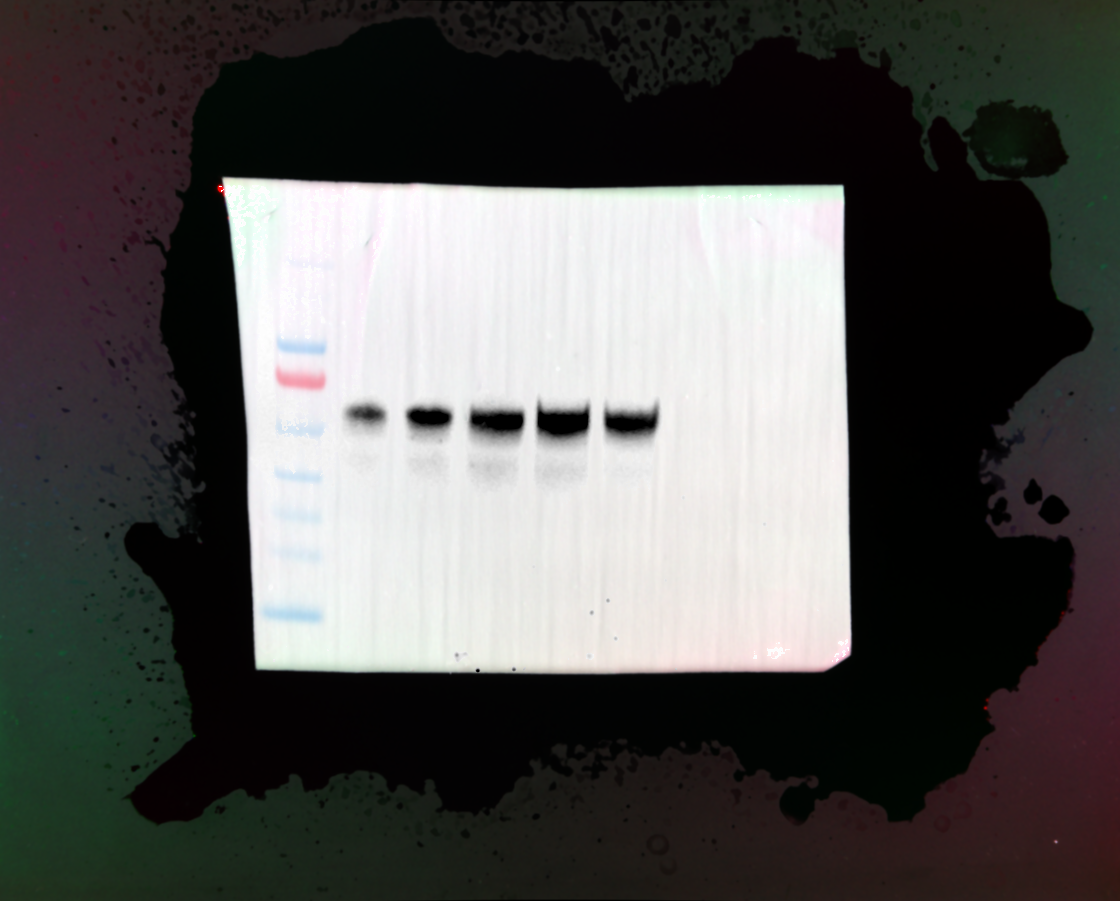

Supplement: Figure 1—figure supplement 6—source data 2. [file elife-85930-fig1-figsupp6-data2.zip › Figure 1 - Figure supplement 6- source data 2/Blots used for Figure 1 - Figure Supplement 6H graph- raw/VOOH_pAkt_3rd replicate.tif]

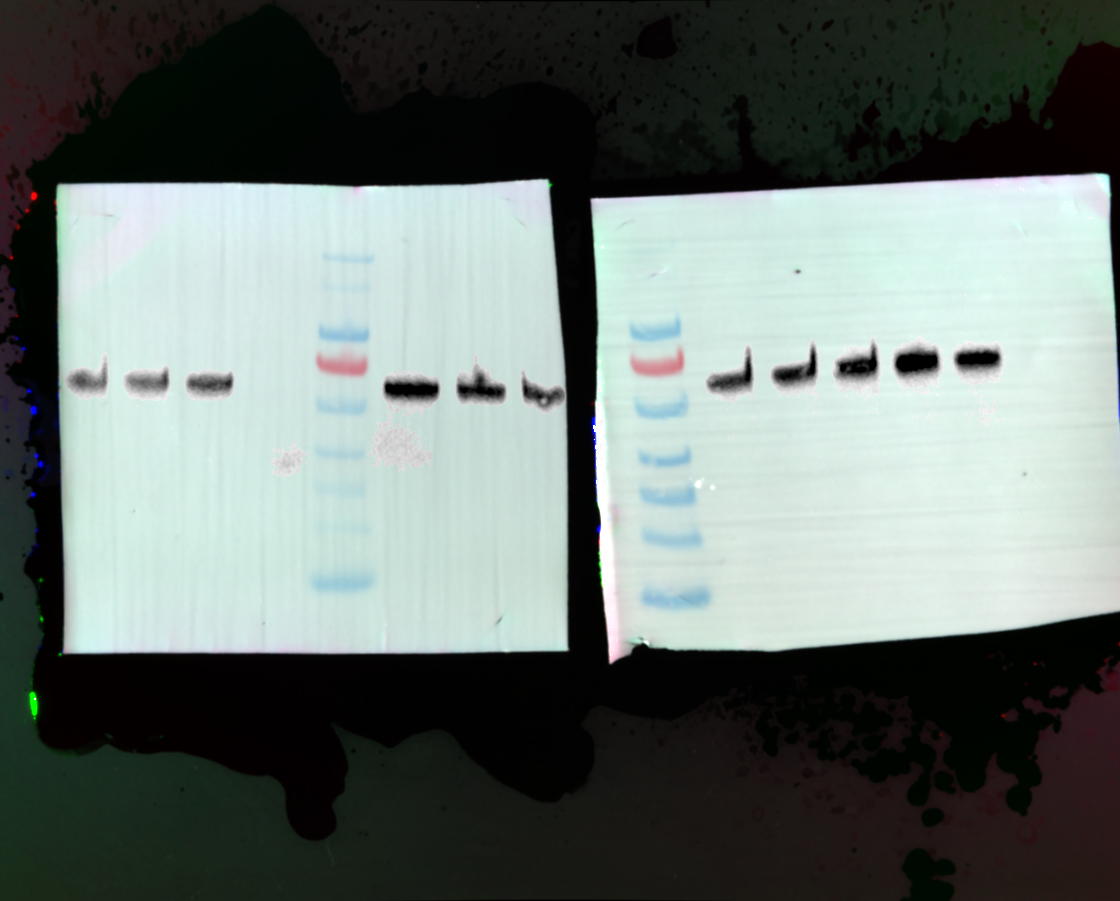

Supplement: Figure 1—figure supplement 6—source data 2. [file elife-85930-fig1-figsupp6-data2.zip › Figure 1 - Figure supplement 6- source data 2/Blots used for Figure 1 - Figure Supplement 6H graph- raw/VOOH_Akt_2nd replicate.tif]

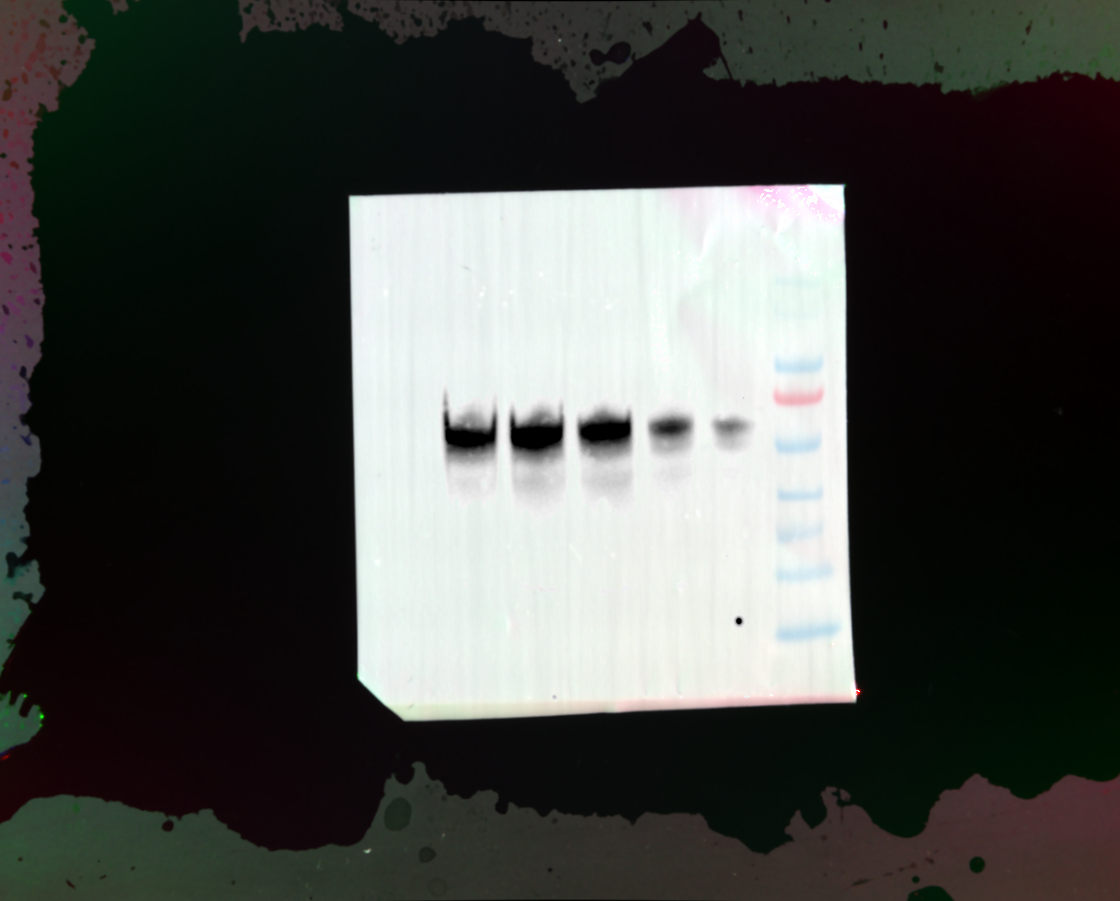

Supplement: Figure 1—figure supplement 6—source data 2. [file elife-85930-fig1-figsupp6-data2.zip › Figure 1 - Figure supplement 6- source data 2/Blots used for Figure 1 - Figure Supplement 6H graph- raw/VOOH_pAkt_2nd replicate.tif]

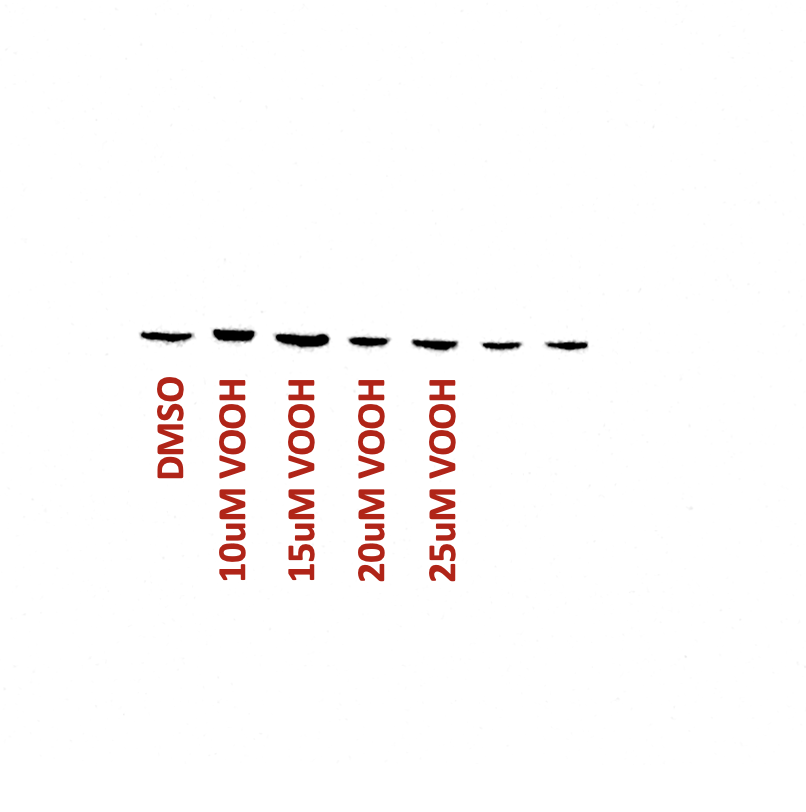

Supplement: Figure 1—figure supplement 6—source data 2. [file elife-85930-fig1-figsupp6-data2.zip › Figure 1 - Figure supplement 6- source data 2/Blots used for Figure 1 - Figure Supplement 6H graph- labelled/VOOH_Akt_3rd replicate_labelled.tiff]

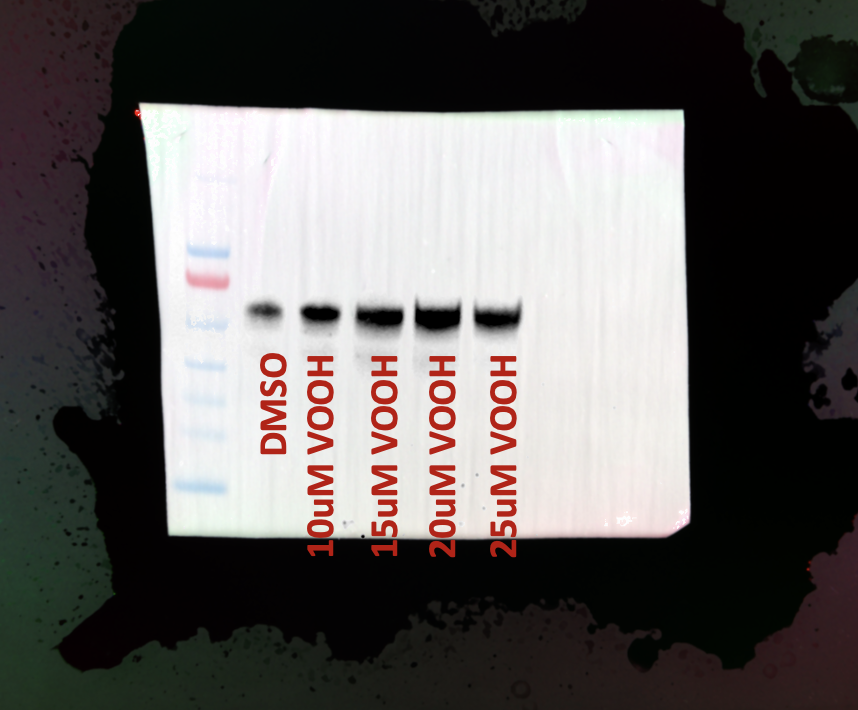

Supplement: Figure 1—figure supplement 6—source data 2. [file elife-85930-fig1-figsupp6-data2.zip › Figure 1 - Figure supplement 6- source data 2/Blots used for Figure 1 - Figure Supplement 6H graph- labelled/VOOH_pAkt_3rd replicate_labelled.tiff]

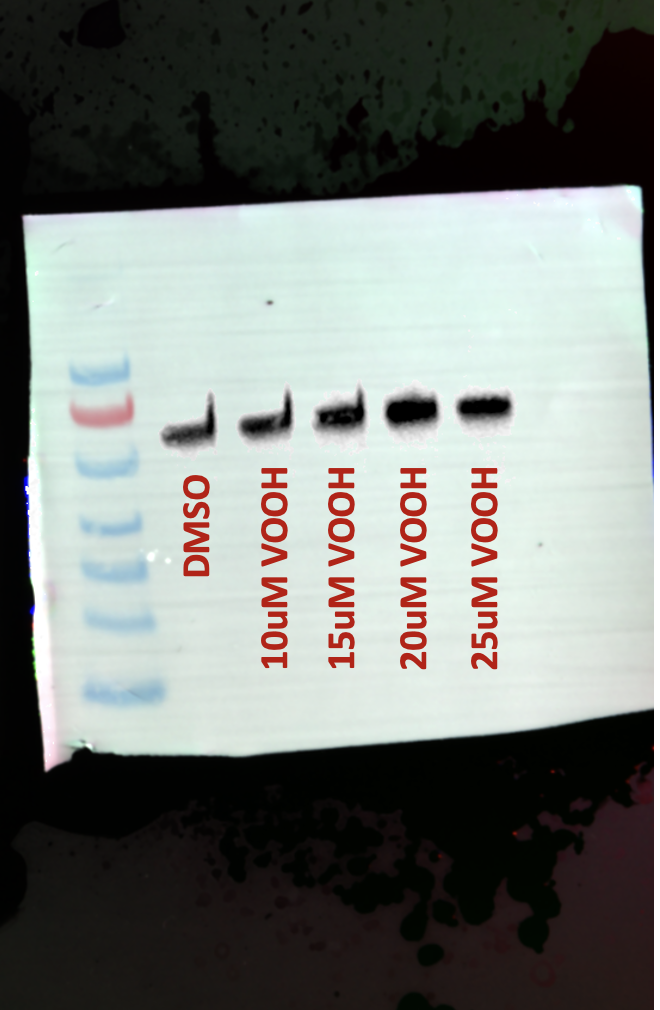

Supplement: Figure 1—figure supplement 6—source data 2. [file elife-85930-fig1-figsupp6-data2.zip › Figure 1 - Figure supplement 6- source data 2/Blots used for Figure 1 - Figure Supplement 6H graph- labelled/VOOH_Akt_2nd replicate_labelled.tiff]

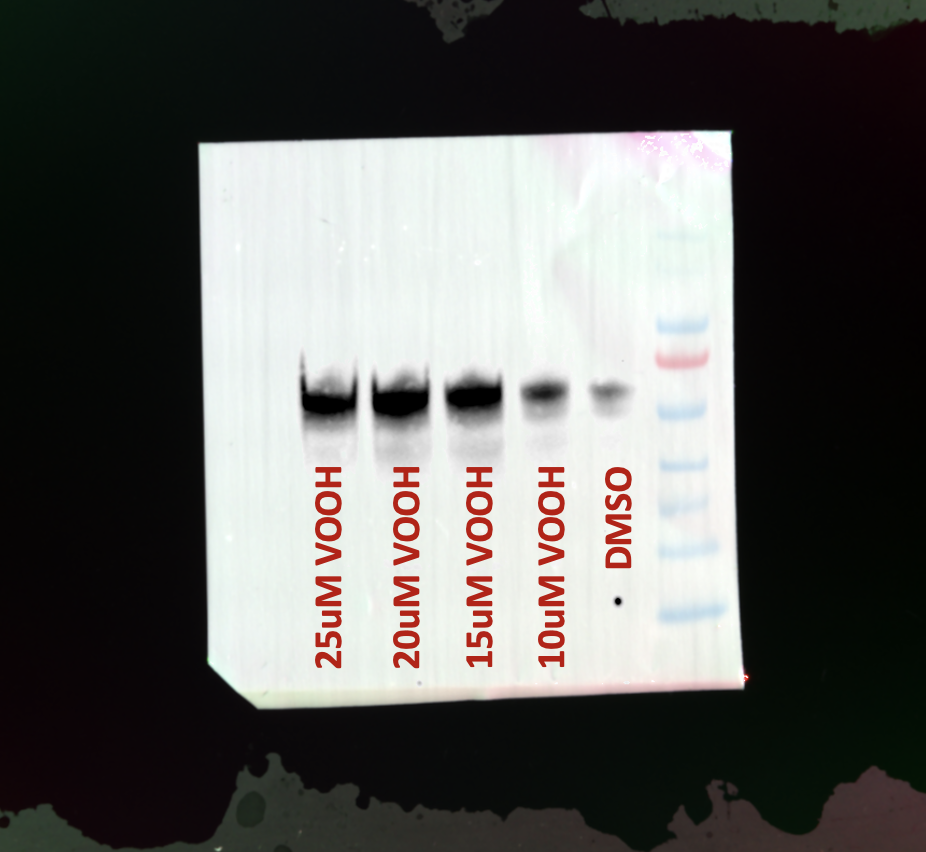

Supplement: Figure 1—figure supplement 6—source data 2. [file elife-85930-fig1-figsupp6-data2.zip › Figure 1 - Figure supplement 6- source data 2/Blots used for Figure 1 - Figure Supplement 6H graph- labelled/VOOH_pAkt_2nd replicate_labelled.tiff]

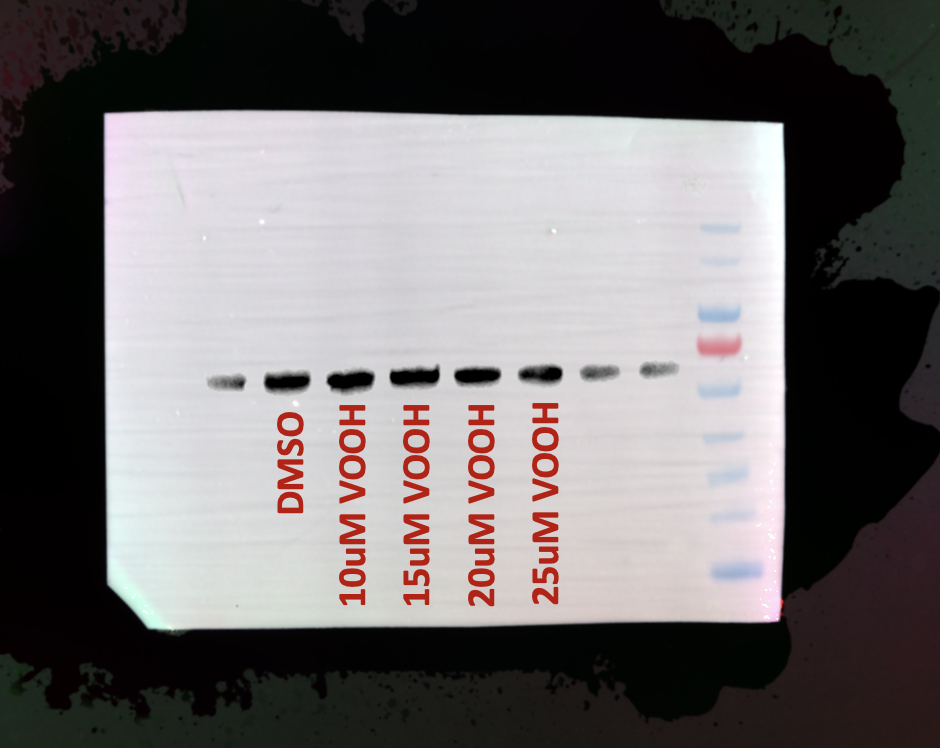

Supplement: Figure 1—figure supplement 6—source data 2. [file elife-85930-fig1-figsupp6-data2.zip › Figure 1 - Figure supplement 6- source data 2/Blots used for Figure 1 - Figure Supplement 6H graph- labelled/VOOH_Akt_1st replicate_labelled.tiff]

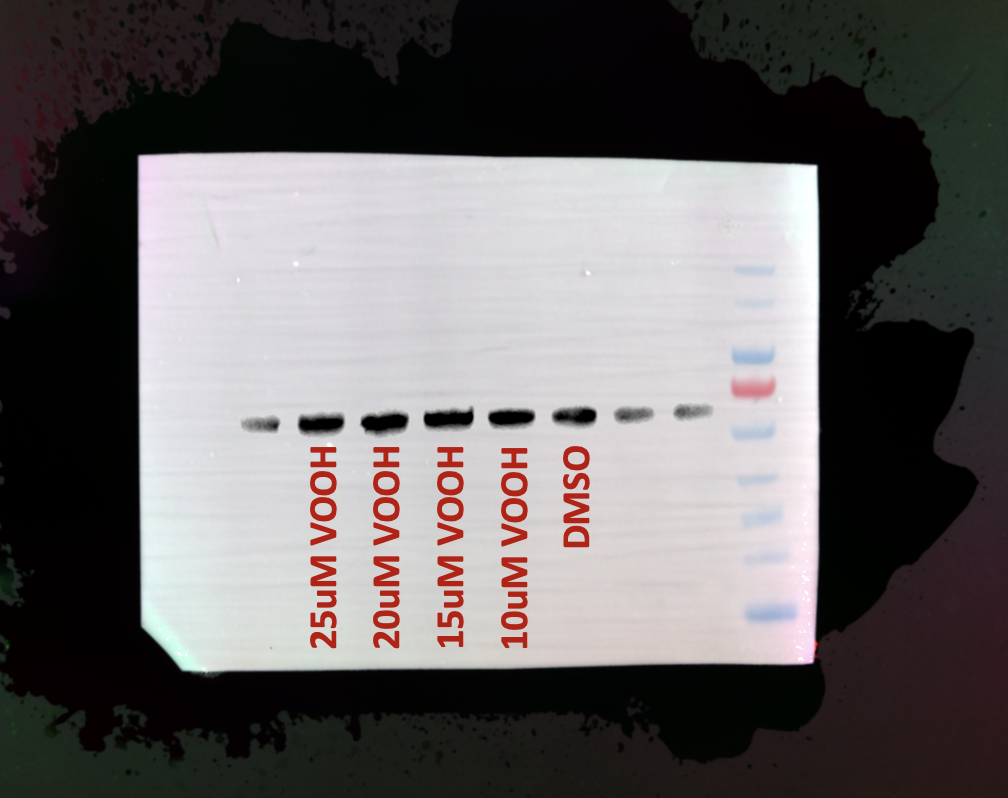

Supplement: Figure 1—figure supplement 6—source data 2. [file elife-85930-fig1-figsupp6-data2.zip › Figure 1 - Figure supplement 6- source data 2/Blots used for Figure 1 - Figure Supplement 6H graph- labelled/VOOH_pAkt_1st replicate_labelled.tiff]

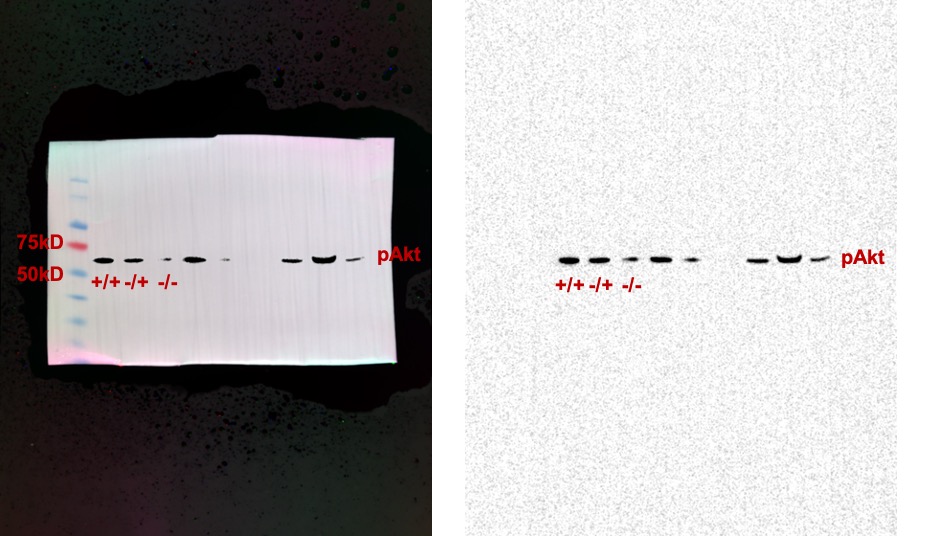

Supplement: Figure 5—source data 2. [file elife-85930-fig5-data2.zip › Figure 5-source data 2/Figure 5A/Blots used for Figure 5A graph- labelled/pAkt_2nd replicate.jpg]

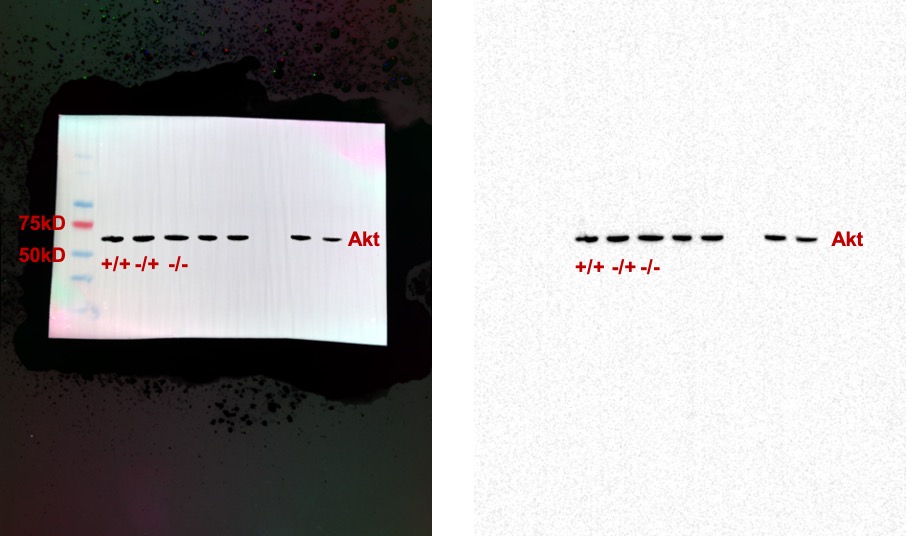

Supplement: Figure 5—source data 2. [file elife-85930-fig5-data2.zip › Figure 5-source data 2/Figure 5A/Blots used for Figure 5A graph- labelled/Akt_2nd replicate.jpg]

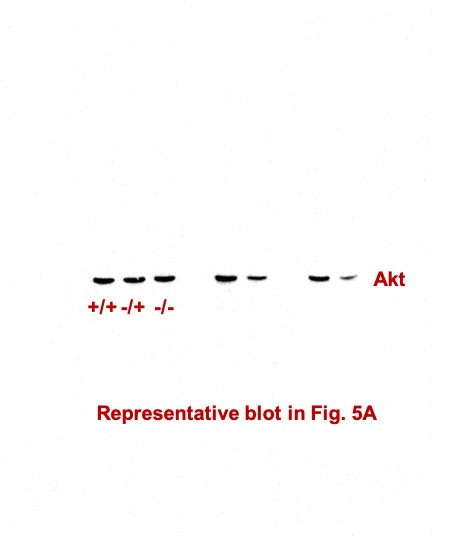

Supplement: Figure 5—source data 2. [file elife-85930-fig5-data2.zip › Figure 5-source data 2/Figure 5A/Blots used for Figure 5A graph- labelled/Akt_3rd replicate.jpg]

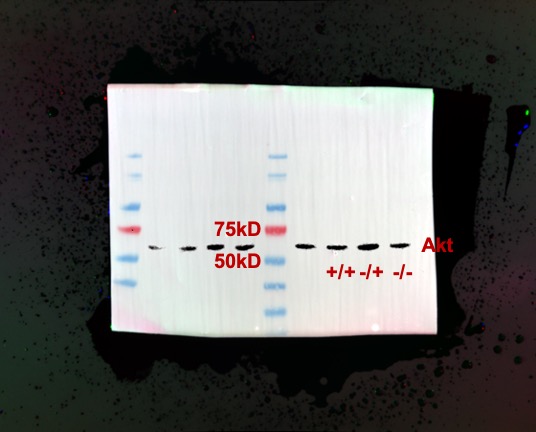

Supplement: Figure 5—source data 2. [file elife-85930-fig5-data2.zip › Figure 5-source data 2/Figure 5A/Blots used for Figure 5A graph- labelled/Akt_1st replicate.jpg]

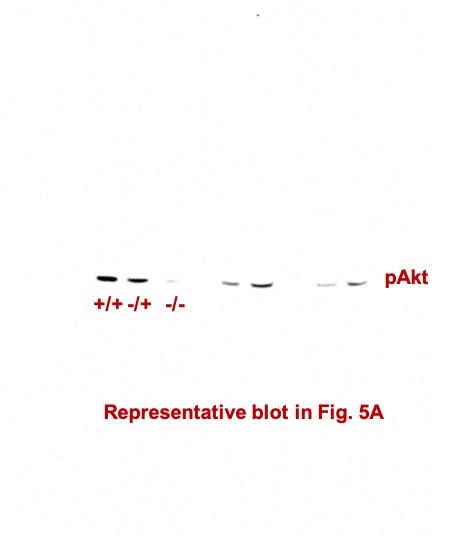

Supplement: Figure 5—source data 2. [file elife-85930-fig5-data2.zip › Figure 5-source data 2/Figure 5A/Blots used for Figure 5A graph- labelled/pAkt_3rd replicate.jpg]

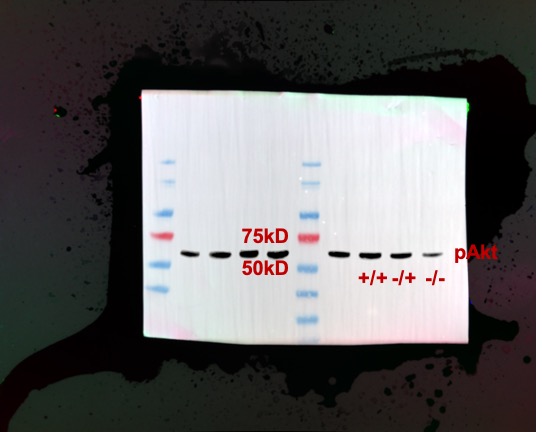

Supplement: Figure 5—source data 2. [file elife-85930-fig5-data2.zip › Figure 5-source data 2/Figure 5A/Blots used for Figure 5A graph- labelled/pAkt_1st replicate.jpg]

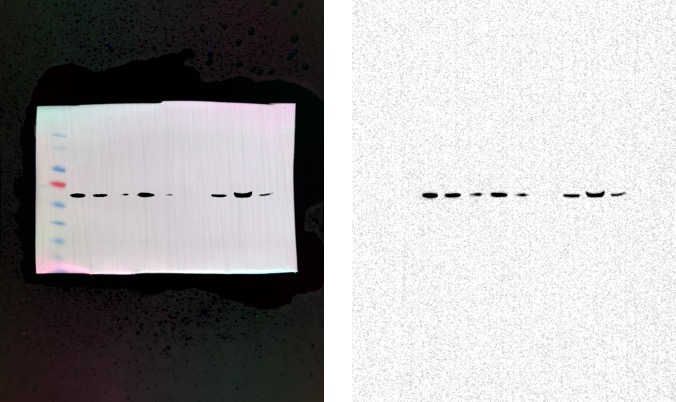

Supplement: Figure 5—source data 2. [file elife-85930-fig5-data2.zip › Figure 5-source data 2/Figure 5A/Blots used for Figure 5A graph- raw/pAkt_2nd replicate.jpg]

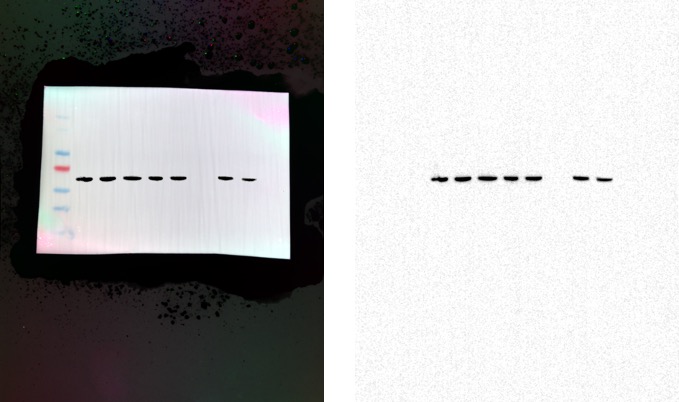

Supplement: Figure 5—source data 2. [file elife-85930-fig5-data2.zip › Figure 5-source data 2/Figure 5A/Blots used for Figure 5A graph- raw/Akt_2nd replicate.jpg]

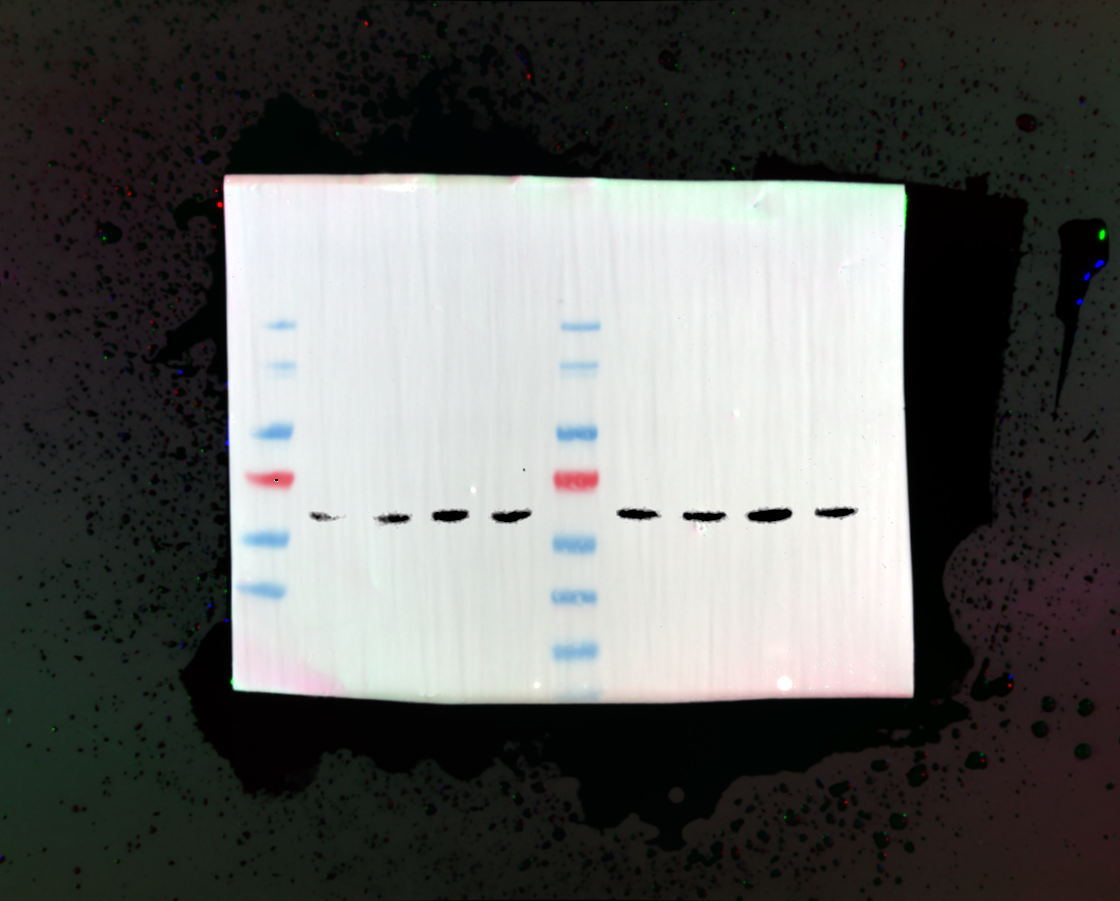

Supplement: Figure 5—source data 2. [file elife-85930-fig5-data2.zip › Figure 5-source data 2/Figure 5A/Blots used for Figure 5A graph- raw/Akt_1st replicate.tif]

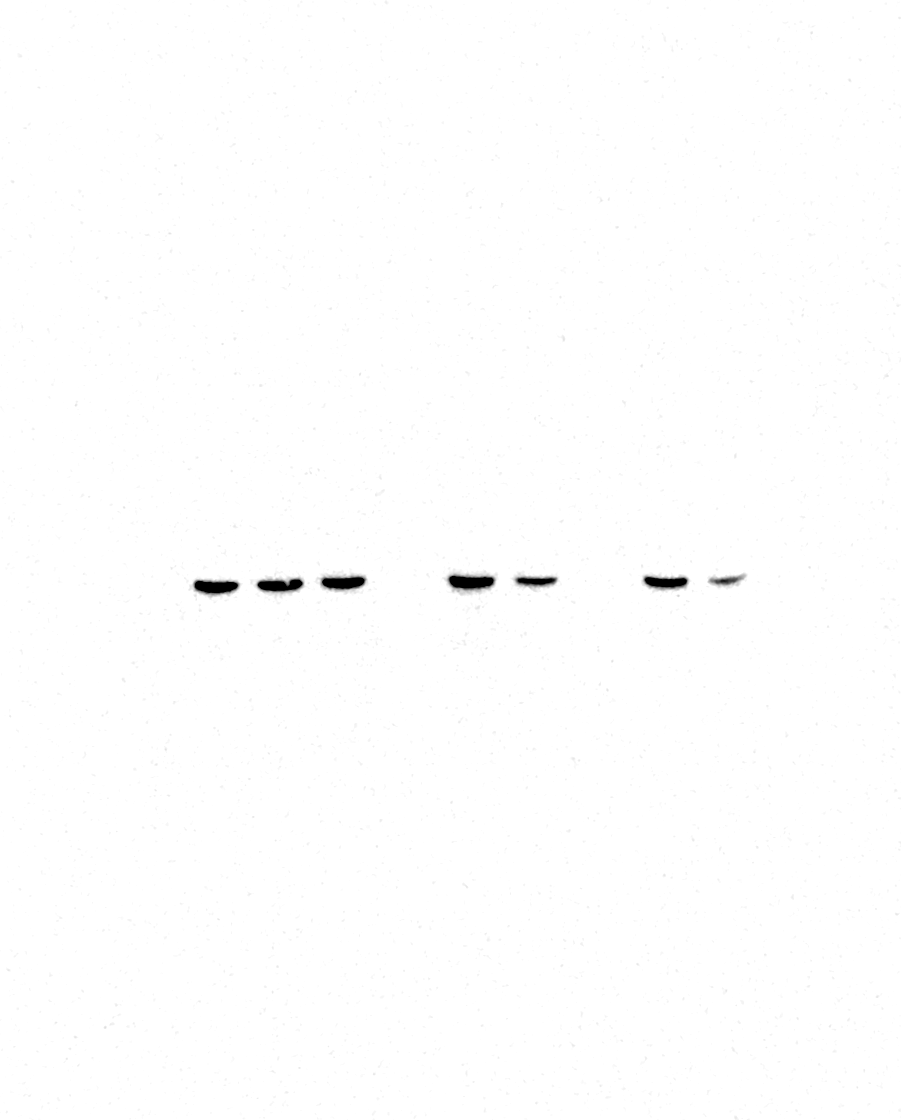

Supplement: Figure 5—source data 2. [file elife-85930-fig5-data2.zip › Figure 5-source data 2/Figure 5A/Blots used for Figure 5A graph- raw/Akt_3rd replicate.tif]

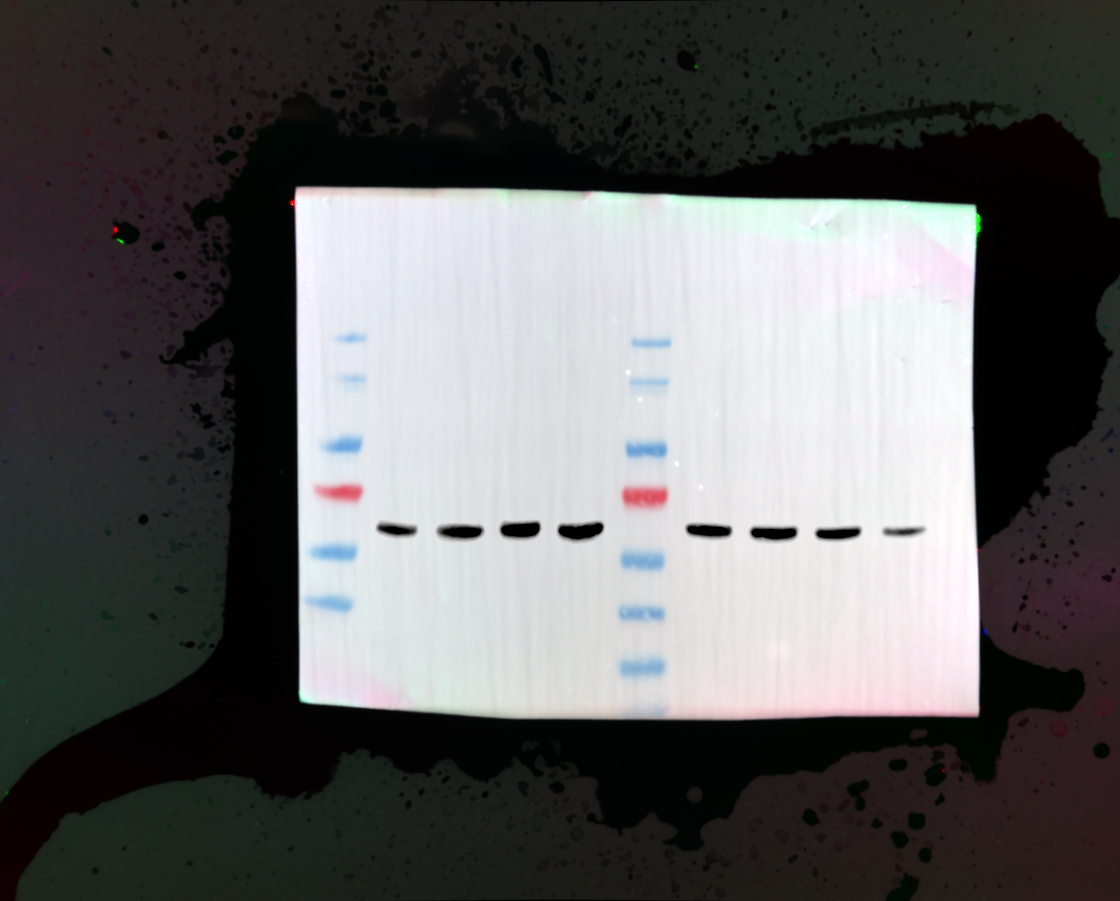

Supplement: Figure 5—source data 2. [file elife-85930-fig5-data2.zip › Figure 5-source data 2/Figure 5A/Blots used for Figure 5A graph- raw/pAkt_1st replicate.tif]

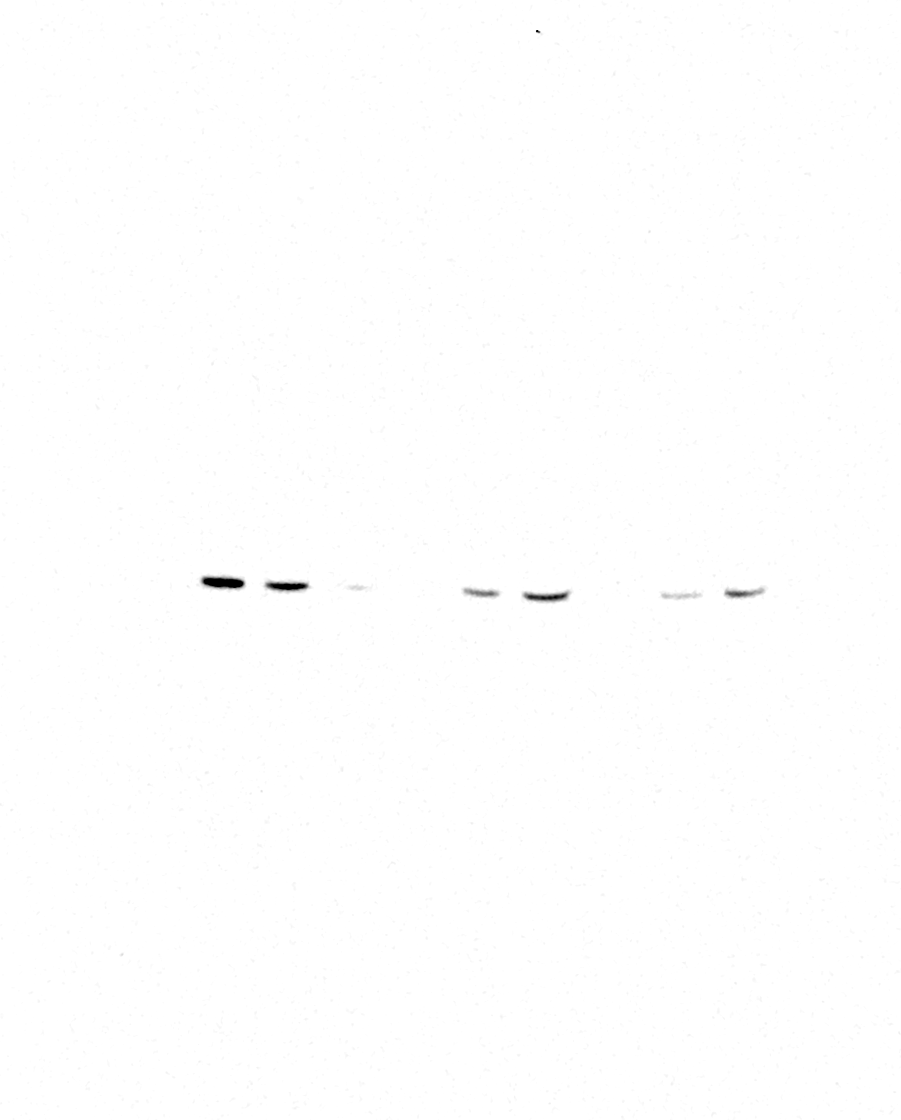

Supplement: Figure 5—source data 2. [file elife-85930-fig5-data2.zip › Figure 5-source data 2/Figure 5A/Blots used for Figure 5A graph- raw/pAkt_3rd replicate.tif]

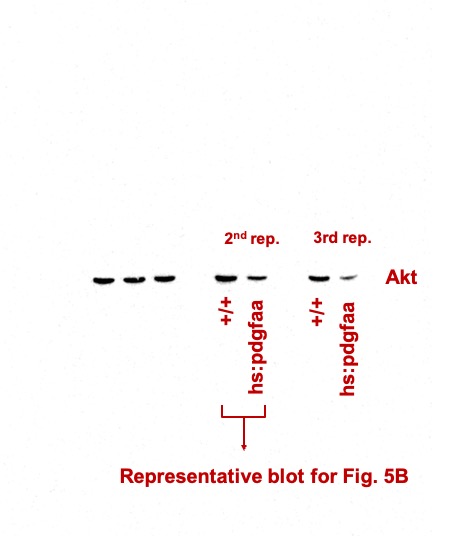

Supplement: Figure 5—source data 2. [file elife-85930-fig5-data2.zip › Figure 5-source data 2/Figure 5B/Blots used for Figure 5B graph- labelled/Akt_2nd_3rd replicate.jpg]

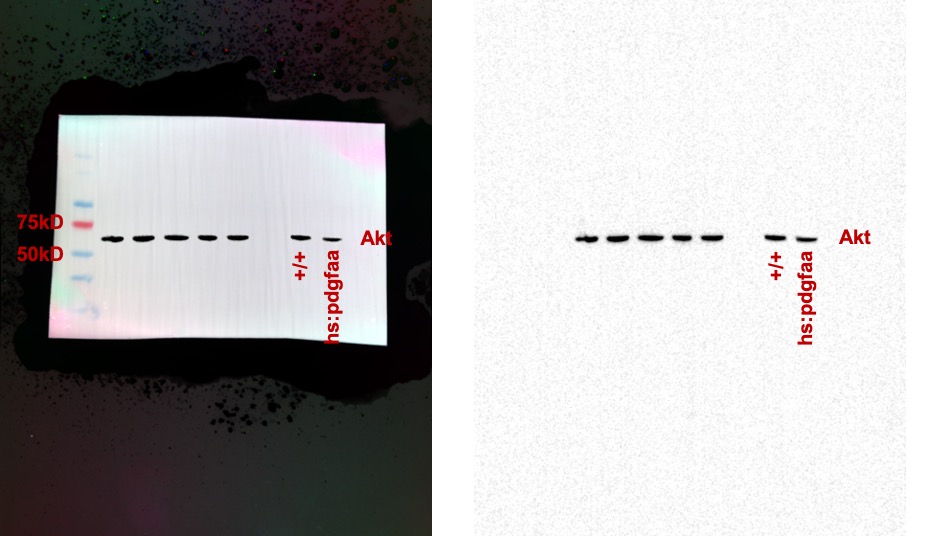

Supplement: Figure 5—source data 2. [file elife-85930-fig5-data2.zip › Figure 5-source data 2/Figure 5B/Blots used for Figure 5B graph- labelled/Akt_1st replicate.jpg]

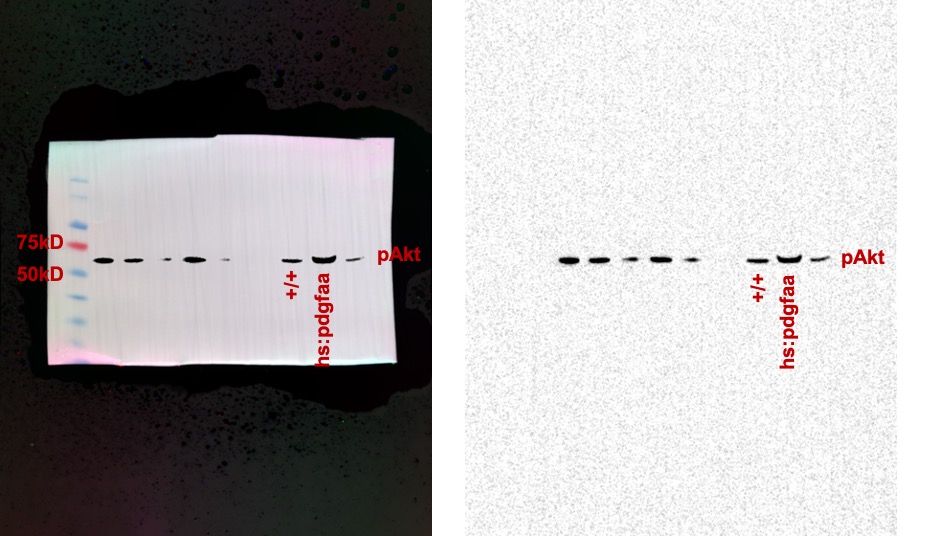

Supplement: Figure 5—source data 2. [file elife-85930-fig5-data2.zip › Figure 5-source data 2/Figure 5B/Blots used for Figure 5B graph- labelled/pAkt_1st replicate.jpg]

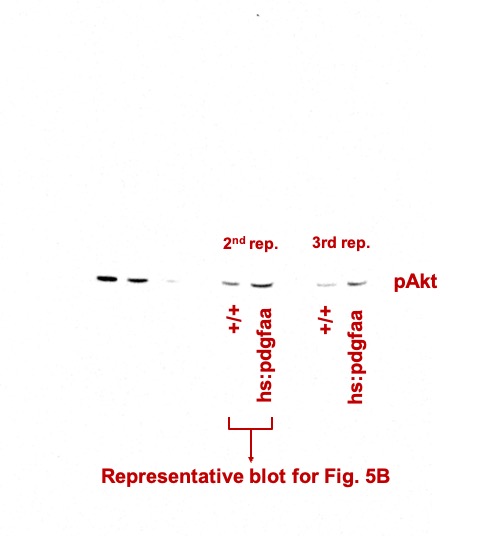

Supplement: Figure 5—source data 2. [file elife-85930-fig5-data2.zip › Figure 5-source data 2/Figure 5B/Blots used for Figure 5B graph- labelled/pAkt_2nd_3rd replicate.jpg]

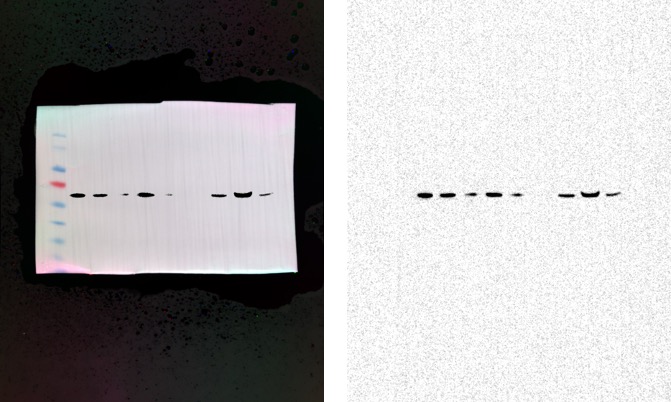

Supplement: Figure 5—source data 2. [file elife-85930-fig5-data2.zip › Figure 5-source data 2/Figure 5B/Blots used for Figure 5B graph- raw/pAkt_1st replicate..jpg]

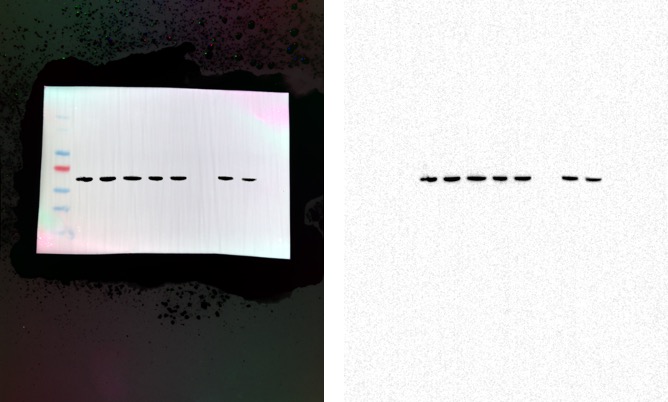

Supplement: Figure 5—source data 2. [file elife-85930-fig5-data2.zip › Figure 5-source data 2/Figure 5B/Blots used for Figure 5B graph- raw/Akt_1st replicate.jpg]
